# A Comprehensive Study of the Cobalt(II) Chelation Mechanism by an Iminodiacetate-Decorated Disaccharide Ligand

**DOI:** 10.3390/molecules30153263

**Published:** 2025-08-04

**Authors:** Cécile Barbot, Laura Gouriou, Mélanie Mignot, Muriel Sebban, Ping Zhang, David Landy, Chang-Chun Ling, Géraldine Gouhier

**Affiliations:** 1 Institut CARMeN UMR 6064, University Rouen Normandie, INSA Rouen Normandie, University Caen Normandie, ENSICAEN, CNRS, FR 3038, INSA Rouen, CNRS, IRCOF, 76821 Mont Saint Aignan, France; cecile.barbot@univ-rouen.fr (C.B.); laura.gouriou2@univ-rouen.fr (L.G.); melanie.mignot@insa-rouen.fr (M.M.); muriel.sebban@univ-rouen.fr (M.S.); 2 Department of Chemistry, University of Calgary, Calgary, AB T2N 1N4, Canada; zhap@ucalgary.ca; 3 UCEIV, UR4492 Université du Littoral Côte d’Opale, Department of Chemistry, 59140 Dunkerque, France; landy@univ-littoral.fr

**Keywords:** cobalt(II) chelation, iminodiacetate ligand, α-maltoside, ITC, speciation diagram, binding constant

## Abstract

We report an investigation on the cobalt(II) chelation mechanism by a modified α-maltoside ligand **9** decorated with two iminodiacetate (IDA) residues on C6,C6′ positions. Herein we uncovered the capacity of this biodegradable ligand to chelate cobalt(II), an ionic metal contaminant in the environment that is used, in particular, in lithium-ion batteries. The interactions between cobalt(II) and synthesized ligand **9** were systematically studied using different analytical methods such as ^1^H and ^13^C NMR, potentiometry, spectrophotometry, ITC, and ICP-AES. We observed a high affinity for the 1:1 complex, one cobalt(II) associated with two iminodiacetate groups, which is 10-fold higher than the 2:1 complex, where each of the two IDA groups interacts alone with a cobalt(II). Taking into account the log ***β_CoL_*** value obtained (≈12.3) with the stoichiometry 1:1, the strength of this complexation with cobalt(II) can be ranked as follows for the most common ligands: IDA < MIDA < NTA < **9** < EDTA < TTHA < DTPA. We further completed a preliminary remediation test with water contaminated with cobalt(II) and recovered cobalt(II) metal using Chelex^®^ resin, which allowed a recycling of the synthetic ligand for future recovering experiments. The results shed light on the great potential of using this synthetic ligand as an effective and green remediation tool.

## 1. Introduction

In the context of energy storage in portable devices and electric vehicles, the demand for lithium-ion batteries (LIBs) has exploded during recent years, thus the extraction and the reuse of their components have become crucial. Indeed, the production of LIBs was approximately 685 GWh in 2024 and will exceed 2035 GWh by 2030 [1]. The recycling industrial process of end-of-life Ni-MH and Ni-Cd batteries is currently well developed and profitable. This is not yet the case for LIBs that mainly use lithium (5–7%), copper (5–20%), cobalt (5–20%) and nickel (5–10%). Only 5% of the LIBs in the landfills are recycled. Contrary to the lithium and nickel market, the cobalt market is under pressure because of uncertainty in cobalt supply from geographical and political aspects [2]. Indeed, the cobalt market will increase by 15 fold in 2030, suggesting an increase in the scarcity of this essential metal. Cobalt(II) is a toxic and expensive key metal used in LIBs as a cathode stabilizer. Reducing the metal’s environmental footprint is a worldwide challenge, thus it is now regulated by legislation rules [3]. The aims are twofold: to decrease the impact on the health of living organisms and to address the scarcity of cobalt in the future. At the same time, the management of strategic metal resources such as lithium and cobalt is fundamental to achieve a circular and sustainable economic model. The composition and shapes of current LIBs vary significantly, making their recycling difficult and very expensive. After sorting, dismantling, grinding, and pre-treatment, two industrial processes are commonly used. The first one is pyrometallurgy, which is an energy-demanding process that heats up the heterogeneous mixture to 1400 °C to melt the crude materials; the process generates slag and greenhouse gases and the extracted metals are used in the steel and superalloys industries. The second one is hydrometallurgy, which is based on a leaching process using strong inorganic acids and organic solvents to selectively recover metals of interest with a higher purity. The second extraction process requires homogeneous inflow at room temperature but is more harmful to the environment than the pyrometallurgy process, because of the effluents created. Indeed, acid leaching causes equipment corrosion, water source contamination, and atmospheric pollution. Consequently, improvement of the recycling processes by developing new tools derived from environmentally friendly and biodegradable materials can be an effective solution, thus new precursors of materials from biomass are ideal to reach the green transition in order to extract metal by filtration or precipitation. Alternative environmentally friendly routes have also been developed using green solvents such as nonionic deep eutectic [4] or ionic liquid [5] or effective organic acids such as citric, oxalic, malic, ascorbic, acetic, lactic, tartaric, aspartic, formic, and iminodiacetic acids [1]. The use of organic acids with low acidity generates no toxic gas during the leaching/recovery process, reducing the harm to the environment. For instance, Keny and collaborators reported the use of iminodiacetic and ascorbic acids as reducing agent to recover metal ions via dissolution at 80 °C in aqueous solution [6]. There are many other aminopolycarboxylic acid ligands, whose pK_a_s and stability constants with cobalt(II) are given in Supplementary Materials (Supporting Information, S2) [7,8,9,10,11,12,13,14,15,16,17], such as (methylimino)diacetic acid (MIDA), triethylenetetramine hexaacetic acid (TTHA), ethylenediamine tetraacetic acid (EDTA), diethylenetriamine pentaacetic acid (DTPA), nitrilotriacetic acid (NTA) and iminodiacetic acid (IDA); these ligands are well known to interact with positively charged metal thanks to their chelating properties. However, strong chelating agents with cobalt(II), such as TTHA (log ***β_CoTTHA_*** ≈ 18–20), DTPA (log ***β_CoDTPA_*** ≈ 19), and EDTA (log ***β_CoEDTA_*** = 16–18), should be avoided because they formed complexes that cannot be easily dissociated, limiting their utilities in the recycling process. We reported herein the synthesis of a new carbohydrate-based ligand and its binding properties to cobalt(II) ion. We have selected IDA as a key component in our ligand design, because IDA is a well-established tridentated unit that shows good chelation to many metal ions such as Ni^2+^, Cu^2+^, Pb^2+^, Hg^2+^, Cd^2+^, Fe^3+^, Zn^2+^, Mg^2+^, and Na^+^. It is essential to control pH in order to promote the metal coordination. The first IDA-cobalt(II) (log ***β_CoIDA_*** ≈ 7) complex was reported in the 1960s, with the two carboxylate groups and the central amine donor forming two five-membered rings. As the binding constants with IDA are generally weaker, it can be used as a chemical separator. To remove cobalt(II) from the medium, IDA has been immobilized on various supports such as polysiloxane [18], polymer [19,20,21], silica gel [22], and graphene [23]. We designed a green adsorbent model based on a disaccharide unit functionalized with two IDA ligands. This oligosaccharide moiety is present in polysaccharides and cyclodextrins scaffolds, which could be used as potential support for a future green extraction process. In previous work, the authors have synthesized a disaccharide chelator functionalized with an IDA ligand, as a cyclodextrin model unit to chelate gadolinium ion for MRI application [24]. In this context, we extended the application of this disaccharide to develop a greener strategy to capture cobalt(II) ion. The ligand is based on the methyl α-maltoside disaccharide functionalized at C6,C6′ positions with two 1*H*-1,2,3-triazole units bearing two IDA residues (Figure 1). Potentially, the two arms can collaborate, completing a coordination sphere for one cobalt(II) ion and act independently, creating coordination spheres for two cobalt(II) ions. The 1*H*-1,2,3-triazole function can also potentially be involved in metal coordination chemistry while the two glucopyranosyl units create a molecular support to enhance the structural rigidity of the complex. On the other hand, the acid and enzyme-sensitive glycosidic linkage of the disaccharide could present a clear advantage over the traditional ligands such as EDTA and DPTA during the metal-recovering processes, while leaving smaller environmental footprints. In 2022, Boland and Stone have reported a kinetic study nickel(II) capture in aquatic environments using a *N*-substituted IDA a trans-1,2-diaminocyclohexane-*N,N,N′,N′*-tetraacetate (two IDA units covalently attached to a 1,2-trans-disubstituted cyclohexane) [25]. The steric bulk at the *N*-substituted position and the presence of a cyclohexane ring reduce the kinetic rate by restricting the rotation of the two hexadentate iminodiacetate moieties. While organometallic coordination depends on multiple factors including ligand structure, ligand/complex rigidity, protonation strength, and steric, electrostatic and electronic interactions, the development of novel, effective and recyclable approaches requires further fundamental research. This work exemplifies an innovative use of biodegradable carbohydrate-based ligands for metal recovery.

Original ^1^H and ^13^C NMR experiments have been performed on the complexes with the ratio cobalt(II):**9** 1:1 and 2:1, confirming the formation of IDA-Co complexes. We conducted the detailed binding studies and determined the stability constants of the complexes using potentiometry, spectrophotometry, and isothermal calorimetry (ITC) and used information to elucidate the mechanism of chelation. Finally, the complexation and release of cobalt(II) ions were tracked by the ICP-AES experiment, and this provides evidence to reveal the potential of using this chelator to capture previous metals.

## 2. Results and Discussion

### 2.1. Synthesis of Ligand ***9***

The synthesis of the disaccharide ligand **9** was described by the authors in five steps using the copper(I)-catalyzed azide-alkyne cycloaddition (“click” reaction) between 6,6′-diazide of α-methyl maltoside **4** and *N*-propargyl iminodiacetate dimethyl ester **7** (Scheme 1) [24]. In summary, a Fisher glycosylation from maltose **1** in methanol led to a methyl glycoside **2** as a 1:1 anomeric mixture (30% yield). After regioselectively converting the two primary hydroxyl groups into the 6,6′-dibromide derivative using *N*-bromosuccinimide/triphenylphosphine in anhydrous *N*,*N*-dimethylformamide (DMF), a per-*O*-acetylation was carried out to afford the crude compound **3,** which was directly substituted by sodium azide to afford the corresponding 6,6′-diazide as an anomeric mixture (α/β ≈ 1:1, ~48% yield over three steps). The α-anomer **4** was obtained in pure form after a further purification by HPLC on a normal-phase silica gel column using a gradient of ethyl acetate -dichloromethane (0→5%) as the eluent. The second reagent was obtained in two steps from commercially available *N*,*N*-iminodiacetic acid **5**. The first step is an acid-catalyzed *O*-methylation to afford the bis(methyl ester) hydrochloride **6** (~100% yield). The salt obtained was then *N*-alkylated with propargyl bromide using *N*,*N*-di*iso*propylethylamine (DIPEA) as a base, providing the corresponding tertiary amine **7** in good yield (65%) after column chromatography on silica gel. The next step was the convergent coupling between the 6,6′-diazido-α-maltoside **4** and the alkyne **7** in acetone; the 1,3-dipolar cycloaddition afforded the desired conjugate **8** at 71% yield after a purification by column chromatography on silica gel. After two sequential deprotection steps by first removing all the *O*-acetates using Zemplén transesterification, saponification to hydrolyze all the methyl esters followed. The pure ligand **9** was obtained (92% yield) by gel filtration on Sephadex LH-20. The purity and identity of ligand **9** were confirmed by ^1^H and ^13^C nuclear magnetic resonance (NMR) spectroscopy and electrospray high-resolution mass spectrometry in the negative ion mode (ESI-HRMS).

### 2.2. Formation of Complexes Cobalt(II):***9***

We next studied the capacity of ligand **9** to chelate and release transition metals using cobalt(II) as a model. To validate this potential of chelation, two complexes have been studied using 1:1 and 2:1 ratios of cobalt(II):**9**, respectively, by adding a solution of CoCl_2_.6H_2_O to ligand **9**.

There are many ways to form cobalt(II) complexes with this maltoside scaffold-based IDA ligand **9**: one of them could take advantage of the two IDA units that can collaborate to form an octahedral coordination sphere to complex one cobalt(II) metal, so a 1:1 cobalt(II):**9** is formed (Scheme 2). In the other one, each of the IDAs unit could act alone to coordinate with one cobalt(II) metal, and each cobalt(II) center is further complemented with three water molecules to form the required octahedral coordination sphere; this would afford a 2:1 cobalt(II):**9** complex (Scheme 2). [Co(IDA)(H_2_O)_2_] crystal structure was reported in the literature in 2020 and deposited in the Cambridge Crystallographic Data Center [26]. Unfortunately, due to structural complexity of the complexes stoichiometry and the strong hygroscopy of the carbohydrate ligand, we were unable to obtain crystal structures.

The identity of the two complexes were, respectively, confirmed by high-resolution mass spectrometry (HRMS) using ESI-TOF (negative mode). For example, for the 1:1 cobalt(II):**9** complex (ligand **9** + cobalt(II)), we observed signature peaks at *m*/*z* 804.1622 and *m*/*z* 826.1437, which, respectively, correspond to the proposed complex C_27_H_36_N_8_O_17_Co as a proton and sodium adducts (calculated *m*/*z* values for [M + H^+^]^−^: 804.1609 and for [M + Na^+^]^−^: 826.1428. For the 2:1 cobalt(II):**9** complex (ligand **9** + 6H_2_O + 2 cobalt(II)), we observed a main peak at *m*/*z* 861.0799, which corresponds to the proposed complex (C_27_H_48_N_8_O_23_Co_2_) that had lost all six coordinated water molecules in mass spectrometer (calculated *m*/*z* for [M-6H_2_O-H^+^]^−^: 861.0784). The MS spectra are rather complex due to the presence of many ions with different charged states and adducts. The other species observed are reported in Table in S5 Supplementary Information.

Preliminary molecular modeling supports the two proposed 1:1 and 2:1 cobalt(II):**9** complexes (Figure 2). As can be seen, the free bond rotations from multiple σ-bonds responsible for the covalent attachment of the two IDA groups (from the C-6 and C-6′ positions) provide abundant freedom for the formation of octahedral coordination sphere, required for the chelation of the cobalt(II) center(s) in both complexes, resulting in the absence of distortion. Both the 1*H*-1,2,3-triazoles attached to adjacent glucopyranosyl units are not involved in the interactions with the metal centers.

### 2.3. Nuclear Magnetic ResonanceStudy (NMR) of Cobalt(II) Complexes

We next attempted to use NMR spectroscopy to study the two 1:1 and 2:1 cobalt(II):**9** complexes. However, several challenges are present using this technique due to paramagnetic nature of the cobalt(II) complexes [27]: (i) the presence of unpaired electrons in paramagnetic metal centers causes significant line broadening, making it difficult to resolve and quantify individual NMR signals; (ii) furthermore, nuclei close to the paramagnetic metal center may experience a strong interactions that their signals become too broad to be detected, creating “blind spheres” around the paramagnetic center; (iii) large paramagnetic shifts can occur, which complicate the interpretation of NMR spectra. These shifts are often temperature dependent and can vary widely; (iv) paramagnetic metal centers lead to shorter relaxation times, which can result in broader signals and increased noise in the spectra.

Despite these limitations, NMR is a useful and powerful tool to characterize paramagnetic compounds, but these challenges necessitate an adaptation of ^1^H and ^13^C NMR experiments to effectively analyze paramagnetic complexes using special protocols and parameters, such as those recently described by M. Lehr et al. [27]. Consequently, in order to characterize the paramagnetic 1:1 and 2:1 cobalt(II):**9** complexes, we implemented and optimized a set of techniques initially reported [27].

^1^H NMR spectra recorded with a large spectral width for both 1:1 and 2:1 complexes, prepared with the same protocol (Supporting Information S1), were very different from those of ligand **9**. They revealed the presence of broad signals between −100 ppm and 150 ppm, proving the interaction between paramagnetic cobalt(II) and **9**. In addition, ^1^H NMR spectra of complexes 1:1 and 2:1 showed significant differences, suggesting that these two complexes do not have the same structures (Figure 3 and Figure 4). The similarity of several signals in the spectra of the two complexes may indicate a difference in stoichiometry, as expected. However, the broadness of the peaks and the overlapping of numerous signals prevent any quantitative analysis, which would have been necessary to confirm the 1:1 and 2:1 stoichiometries of the complexes.

^13^C NMR spectra confirmed these results (Figure 5). No signal was observed outside the range of conventional ^13^C chemical shifts; however, a significant line broadening was observed in the spectra of the complexes compared to those of pure ligand **9**, supporting the formation of the paramagnetic cobalt(II):**9** complexes. Due to the lack of relevant results in 2D NMR, caused by a weak signal-to-noise ratio resulting from the paramagnetic effect of cobalt(II), the assignment of ^1^H (and ^13^C) signals could not be achieved, hindering a precise structural elucidation.

Very few cobalt(II) NMR experiments were reported in the literature [27]. The NMR conditions implemented for this study showed that the two complexes of cobalt(II):**9** with 1:1 and 2:1 ratios occurred via very different binding interactions, as evidenced by the very different spectra in the expanded regions of the chemical shifts.

### 2.4. Protonation Studies of the Disaccharide Ligand ***9***

#### 2.4.1. Protonation Studies of the Ligand **9** by Potentiometric Titration

In order to evaluate the binding affinity occurred in the complexes of cobalt(II):**9**, a protonation study was performed by potentiometric titration to obtain the species diagram. The structure of the disaccharide ligand **9** contains four carboxylate residues and two tertiary amine centers. Depending on the pH of the media, it can exist in different protonation states similar to another already published disaccharide ligand [28].

Based on the well-known pK_a_ values for acetic acid (~4.76) and triethylammonium (~10.75) and a literature report on the pK_a_ of the protonated 1,2,3-triazolium compound, *N*-methyl-1,2,3-triazolium (~1.25) [29], we can safely ignore the protonation of the two 1,2,3-triazole rings in the disaccharide **9**, since it only becomes relevant at very acidic pH. Thus, in less acidic solutions, the ligand **9** contains essentially six prominent protonation sites that include the two tertiary amine centers and four carboxylates.

To facilitate the discussion of our potentiometric titration results, the following notations were adopted **L** to represent the form of a ligand **9** with a full deprotonation of all the above sites, **LH_2_** represents the species with essentially both tertiary amines protonated while the remaining four carboxylate groups deprotonated; **LH_6_** represents the form of the disaccharide ligand **9** with all sited protonated (Scheme 3). Other notations will be used accordingly to designate intermediate deprotonation states.

The protonation constant *β_h_* for the following equilibrium is defined by Equation (1), where **L** represents the fully deprotonated form, and **H** is the proton (charges are omitted).

(1) $${L+hH\;⇌\;LHh;\;\beta }_{h}=\frac{\left[{LH}_{h}\right]}{{\left[H\right]}^{h}[L]}$$

As dissociation constants, *K_a_* values are commonly defined by:

(2) $$K_{a}=\;\frac{\left[H][{LH}_{h-1}\right]}{\left[LH_{h}\right]}$$

It is evident that:

(3) $${pK}_{a}=\log\beta_{h}-log\beta_{h-1}$$

As shown in Figure 6 Left, from the potentiometric curves of ligand **9** alone, it is evident that each excess mol of HCl added needed to be neutralized by the same equivalent of NMe_4_OH, in the full pH range. Table 1 summarizes all the protonation constant values of **9** determined by titration curve refinement with HYPERQUAD suite [30], together with the corresponding acidity constants. All the pK_a_ values were ascribed to the deprotonations of the carboxylate functions, followed by ammonium functions when increasing the base addition. The last carboxylate group to be deprotonated, named **LH_3_**, had a pK_a_ (**LH_3_**/**LH_2_**) equal to 2.47. The pK_a_ values greater than 7 could be attributed to the formation of ammonium moieties, which is in agreement with the known values for these groups (**LH_2_**/**LH**: 7.52–7.60; **LH**/**L**: 8.44–8.49).

The obtained *pK_a_* values are in accordance with the literature for iminodiacetic acid (IDA) in an aqueous medium [11], and those from the ethylenediamine acid-like ligand based on the sucrose scaffold as explained in our precedent publication [28].

From the species diagram of the ligand **9**, in Figure 6 Right, elaborated with the HySS program [31], between pH 4 and 6, **LH_2_** was the predominant form with all the nitrogen protonated and all the carboxylate groups deprotonated. At physiological pH, the majority of species would be **LH_2_** in equilibrium with **LH**.

#### 2.4.2. Protonation Studies of the Ligand **9** by Spectrophotometric Titration

To evaluate the protonation constants of the ligand **9**, spectrophotometric titrations were also conducted manually by adding increasing volume of 0.1 M NMe_4_OH to solutions containing disaccharide in extra-HCl. After each addition of base, and waiting for reaching equilibrium, pH was measured and UV–visible scan was recorded by means of a fiber optic probe dipping into the cell (Figure 7).

This latter ligand alone absorbs UV–visible radiation between 200 and 300 nm as can be seen in Figure 7. Therefore, we have chosen to present results of **9** alone at 250 nm wavelength. At this wavelength, absorbance increased linearly, over pH 6, with the concomitant deprotonation of amino groups. Effectively, the molar absorbance coefficient is 104.4 mol^−1^ L cm^−1^ for **LH_2_**, increasing to 278.4 and 333.6, respectively, for **LH** and **L** species, respectively, as reported in Table 2. Over pH 10, the ligand was fully deprotonated, absorbance, therefore, remained constant.

Refinement of the absorption scans, in Hypspec2014 software, represented in Figure 8, were conducted to evaluate protonation constants of **9**. Results were in accordance with those obtained after refinement of the potentiometric titrations, with HYPERQUAD software, as reported in Table 1.

Calculated distribution curve was similar to those obtained by the potentiometric method (see Figure 6 Right).

### 2.5. Complexation Studies of the Cobalt(II) with the Disaccharide Ligand ***9***

#### 2.5.1. Complexation Studies of Cobalt(II) with the Ligand **9** by Potentiometric Titration

As discussed above, the ligand **9** forms mononuclear and dinuclear complexes in aqueous solution in the presence of cobalt(II) ions. Formation of this type of complexes was confirmed by calculations based on potentiometric, spectrophotometric and ITC measurements.

Each potentiometric or spectrophotometric cell was previously bubbled with argon gas to prevent possible oxidation of cobalt(II) with oxygen from air. The procedure efficiency has been checked in this way: at the end of the titration of the solution of cobalt(II) alone (without disaccharide), a blue–green precipitate occurred due to excess hydroxide ions, which was characteristic of the formation of Co(OH)_2_. Nevertheless, after transferring this precipitate into an open pillbox, leaving it under air, by giving up the argon gas, this latter oxidized effectively in several hours in a dark-brown precipitate due to oxidation of cobalt(II) into cobalt(III), forming Co(OH)_3_ precipitate. This emphasize the interest of using an inert gas to prevent cobalt(II) oxidation. For ITC measurements, all the solutions were degassed before each calorimetric titration.

To gain a deeper understanding of the complex formed between the ligand **9** and cobalt(II) in an aqueous solution, we next determined the stability constant using potentiometric titration. A series of solutions containing 0 to 2.36 equivalents of cobalt(II):**9** was prepared by maintaining the initial concentrations of **9** (5.3 × 10^−4^ M) and HCl (3.64 × 10^−3^ M) constant. Similarly, to each prepared solution, potentiometric titrations were performed by adding a titrant solution of tetramethylammonium hydroxide (0.05 M). The pH of the solution was recorded.

With **M** being the metal ion, **L** being the ligand, and **H** being the proton, the stability constants of the complexes with the general formula $M_{m}L_{l}H_{h}$ are expressed by the following equations (the charges are omitted):

(4) $$mM+lL+hH\;⇌M_{m}L_{l}H_{h};\;\beta_{mlh}=\;\frac{\left[M_{m}L_{l}H_{h}\right]}{{\left[M\right]}^{m}{\left[L\right]}^{l}{\left[H\right]}^{h}}$$

Thus, the stepwise protonation constant *K_mlh_* can be defined similarly to the case of the free ligand, as follows:

(5) $$K_{mlh}=\;\frac{\left[M_{m}L_{l}H_{h}\right]}{\left[H][M_{m}L_{l}H_{h-1}\right]}$$

Analogously,

(6) $${\log K_{mlh}=\log\beta_{mlh}-log{\;\beta }_{mlh-1}=-pK}_{d,mlh}$$

where *K_d_* is the dissociation constant, which can be assimilated to *K_a_*, the acidity constant when considering the protonation of the ligand or the complex. In case of hydroxide ion, the species of hydroxylated complex formed (with h<0) are treated taking into account the self-ionization equilibrium:

(7) $$H^{+}+{OH}^{-}⇌H_{2}O\;\;\;\;\left[OH\right]=K_{w}[H{]}^{-1}$$

For the equilibrium between metal and hydroxide for instance:

(8) $$mM+lL+hOH\;⇌M_{m}{L_{l}(OH)}_{h}{\;\;\;\;\;\;\beta }_{h}=\;\frac{\left[M_{m}L_{l}{\left(OH\right)}_{h}\right]}{{\left[M\right]}^{m}{\left[L\right]}^{l}{\left[OH\right]}^{h}}$$

$$\beta_{h}=\frac{\left[M_{m}L_{l}{\left(OH\right)}_{h}\right]}{{\left[M\right]}^{m}{\left[L\right]}^{l}{K_{w}}^{h}[H{]}^{-h}}$$

$$\beta_{h}{K_{w}}^{h}=\frac{\left[M_{m}L_{l}{\left(OH\right)}_{h}\right]}{{\left[M\right]}^{m}{\left[L\right]}^{l}[H{]}^{-h}}$$

$$\beta_{h}^{*}=\beta_{h}{K_{w}}^{h}=\frac{\left[M_{m}L_{l}{\left(OH\right)}_{h}\right]}{{\left[M\right]}^{m}{\left[L\right]}^{l}[H{]}^{-h}}$$

The concentration of the hydroxide ion is now related to the concentration of H^+^. In the HYPERQUAD suite, this convention on the formation constants of hydrolyzed species will appear on a different scale (noted log β^*^) than those reported in the literature (log *β*). The relationship between the two is:

(9) $$\log\beta_{h}^{*}={\log\beta }_{h}+h\log K_{w}\;\;$$

Output stability constants obtained for ligand **9** species have been used, as fixed parameters, to treat data for fitting cobalt(II) binding constants from potentiometric and spectrophotometric experiments. The obtained titration curves of the complex formed between the disaccharide ligand **9** and cobalt(II) are shown in Figure 9 and the calculated log *β_mlh_* and log *K_mlh_* reported in Table 3. For each solution, the pH slowly increased with the addition of the base; however, the pH of the solution with the greatest cobalt(II):**9** ratio increased the slowest when the same amounts of the base were added. This can be explained by the fact that during the complexation of cobalt(II), protons were released from the protonated sites of the disaccharide ligand **9**, acidifying the medium. At the beginning of the titrations, for acidic pH, free cobalt(II) prevailed. Fitting the spectrophotometric titrations curves (see infra) have necessitated the incorporation of mono and dinuclear protonated complex species forms, from pH 2 (beginning pH for spectrophotometric titrations, slight below that of potentiometric experiments), **CoLH_2_** and **Co_2_LH_2_**, followed by **CoLH** and **Co_2_LH** at pH between 3 and 4. Then, **CoL** and **Co_2_L**. For the highest pH, species on the form **CoLH_−1_**, **CoLH_−__2_** or **Co_2_LH_−1_** and **Co_2_LH_−2_** were also incorporated in the model. **CoLH_−1_**, for instance, from its notation, could come from a coordinated water molecule or from an uncoordinated nitrogen. A mixture of mono and dinuclear complexes were observed for the three studied ratios cobalt(II):**9**, the proportion of dinuclear complexes increasing with the cobalt(II) amount, the concentration in ligand **9** remaining constant.

In case of ratio 1:1, free cobalt(II) ions were observed until pH 5, essentially at acidic pH. Above this pH, all the cobalt(II) ions were complexed by the ligand **9**. This complexation process began at extremely low pH with the appearance of **CoLH**, **Co_2_LH_2_** and **Co_2_LH**, in low proportions, at pH between 2 and 4. The coordination process began effectively at low pH, with the deprotonation ${LH}_{3}+Co⇌{CoLH}_{2}+H$ and subsequent deprotonation of **CoLH_2_**, ${CoLH}_{2}⇌CoLH+H$, with log *K*_112_ being equal to 2.6–3.2, which corresponds to the deprotonation of one of the carboxylic groups of iminodiacetate (log *K*_013_ = 2.1–2.5) after binding of cobalt(II) ion.

The complexation on the form of the dinuclear species **Co_2_LH**, still at very low pH, could be interpreted with the deprotonation ${{{Co}_{2}LH}_{2}⇌Co}_{2}LH+H$ with log *K*_212_ being equal to 3–3.1. Then, deprotonation of **Co_2_LH** to **Co_2_L**, ${{Co}_{2}LH⇌Co}_{2}L+H$ with log *K*_211_ similar to log *K*_212_, taking into account the difficulty to evaluate the *pK_a_* at these low pH. These values are also close to that of log *K*_013_, which suggests that carboxylic groups are essentially implicated in the complexation at these low pH. As shown in Figure 6, at pH 5, the speciation of the ligand alone has **LH_2_** as the predominant species (98.8%) that corresponds to the form containing two protonated tertiary amines and four fully deprotonated carboxylate groups. At the same pH, for the ratio 1:1, the speciation diagram, shows that the aqueous solution is a mixture of approximately 42.8% **CoL**, 22.2% **LH_2_**, 19.9% **Co_2_LH_−1_**, 12,7% **CoLH**, and 1.9% **Co_2_L**, relatively to ligand **9** or relatively to cobalt(II) introduced: 42.8% **CoL**, 38.8% **Co_2_LH_−1_**, 12.7% **CoLH**, 3.8% **Co_2_L**, and 0.05% **Co**.

The equilibrium to consider at this pH, from the **LH_2_**, which is the predominant form of the disaccharide alone, at pH 5 should be:

$${LH}_{2}+Co⇌CoL+2H$$

$${LH}_{2}+2Co⇌{Co}_{2}LH_{-1}+3H$$

$${LH}_{2}+Co⇌CoLH+H$$

$${LH}_{2}+2Co⇌{Co}_{2}L+2H$$

At pH 6, the majority species are a mixture of 71.5% **CoL**, 11.0% **Co_2_LH_−1_**, and 10.9% **LH_2_** and to a small extent 3.9% **CoLH_−1_** and 2.0% **CoLH** relatively to ligand **9** or relatively to cobalt(II) introduced: 71.5% **CoL**, 22% **Co_2_LH_−1_**, 3.9% **CoLH_−1_**, 2% **CoLH**, and 0.05% **Co**. At pH 7.3, **CoL** and **CoLH_−1_** with approximately 46% of each species in equilibrium. **Co_2_LH_−1_** and **Co_2_LH_−2_**, sharing the last 8%. This pH is near the pH of deprotonation of the first nitrogen of **LH_2_**. It is possible that a rearrangement of the structure of the complex occurred at this pH with, the participation of nitrogen in the coordination sphere of cobalt(II), for more basic pH. Beyond pH 9, **CoLH_−1_** and **CoLH_−2_** are the predominant species. The disaccharide is almost completely deprotonated and no more protons was exchanged during the formation of the 1:1 stoichiometry of cobalt(II):**9** complex.

Comparing ratios 1:1, 1.5:1, and 2:1, dinuclear complexes prevail with the increasing cobalt(II) amount, with a gradual preponderance of dinuclear species over mononuclear in case of 2:1 ratio. Looking at the stability constants, an analogy could be performed. The stability constants for the formation of **Co_2_LH** and **CoLH_2_** have quite the same values. They are also similar for the couple **Co_2_L**/**CoLH**, **Co_2_LH_−1_**/**CoL** and **Co_2_LH_−2_**/**CoLH_−1_**. Each time a second cobalt(II) forms a dinuclear complex with **9**, a supplementary proton is liberated. The two complexation sites in **9** being equivalents, this explains the similarity of the stability constants for the different couples described previously. Considering mononuclear species, log *K*_112_ is in the same order as the log *K*_013_. As explained before, when forming **CoLH** from **CoLH_2_**, we can reasonably explain the process with the liberation of one proton from one carboxylic group. The same conclusion can be reached when forming **CoL** from **CoLH**, log *K*_111_, approximately 4.5, is characteristic from deprotonation of carboxylic acids. We can assume that **CoL** contains all the carboxylic acid groups deprotonated and the nitrogen protonated. So that the formation of **CoLH_−1_** from **CoL** deprotonates the first of the two nitrogen atoms and the formation of **CoLH_−2_** from **CoLH_−1_** deprotonates the second one. These deprotonations take place at pH higher than pH 7, similarly as the deprotonation of the nitrogen group for the ligand **9** alone, with log *K*_11-1_ and log *K*_11-2_ values in accordance with that from nitrogen deprotonation Upon the addition of one more mole of cobalt(II), the ratio cobalt(II):**9** becomes 2:1. **Co_2_LH_−1_**, now, prevailed at pH 6 whereas, for the ratio 1:1, it was **CoL**; and **Co_2_LH_−2_** prevailed at pH 9–10, whereas, for the ratio 1:1, it was **CoLH_−1_**.

We have observed previously that |log *K*_21-1_| ≈ |log *K*_111_| and that |log *K*_21-2_| ≈ |log *K*_11-1_|, which explains similar acidity and the inversion observed when the ratio gradually increases from 1:1 to 2:1. For a stoichiometry of cobalt(II):**9** equal to 1.5 and 2:1, a white precipitate was observed over pH 8.1 or 8.8, respectively, in the two experiments, potentiometry and spectrophotometry, certainly due to precipitation of the complex on the form **Co_2_LH_−__2_**. The color of the precipitate is different from that of cobalt(II) hydroxide, which is blue, in the absence of the ligand **9**. The stability constant of cobalt(II) hydroxide species alone, **CoH_−__1_** and **CoH_−__2_**, were introduced in the model but do not participate (to be convinced, their stability values were kept constant), as it can be checked in the speciation diagrams. Also, **CoH_−__2_** was not observed into the cell at elevated pH.

In the literature, the stability constants of the cobalt(II) with iminodiacetate ligand (IDA) was reported for the stoichiometries cobalt(II):IDA 1:1 and 2:1 equal to approximately 6.95 and 12.3 [7] or 6.54 and 11.95 [36], respectively. The disaccharide **9** carries two iminodiacetate ligands immobilized on a α-maltoside disaccharide bringing a triazole moiety. This environment reduces the mobility of the complex structure and consequently modify the parameters generating for the stoichiometries cobalt(II):**9** 1:1 and 2:1 binding constants of 12.3 and 16.5, respectively. Taking into account the log ***β_CoL_*** value obtained for the ligand **9** with the stoichiometry 1:1, the strength of the complexation with cobalt(II) can be classified in the following orders: IDA (≈7) < MIDA (≈7.6–8.5) < NTA (≈10) < **9** (≈12.3) < EDTA (≈16–18) < TTHA (≈18–20) < DTPA (≈19). Consequently, the immobilization of the IDA on the green scaffold generates higher binding constant that IDA alone improving its binding affinity to cobalt(II) metal. Metal–ligand complexes with relatively low stability constants are readily biodegradable, whereas those forming stronger complexes (EDTA, TTHA, DTPA) are relatively resistant and persists in the environment [37,38]. The IDA-modified disaccharide **9** combines a good binding constant to be an efficient chelator and a greener ligand than EDTA. Indeed, IDA and the disaccharide are both known to be biodegradable. This improvement clearly shows its potential as remediation agent for trapping and also for releasing cobalt(II).

#### 2.5.2. Complexation Studies of Cobalt(II) with the Ligand **9** by Spectrophotometric Titration

Cobalt(II) solutions did not absorb at the concentration of this study (2.37 × 10^−3^ M) on the entire wavelength at all pHs. Effectively, the ${Co(H_{2}O)}_{6}^{2+}$ aqua-ion is known to have a maximum absorption band at 512 nm, with a low molar absorption coefficient, ε, equal to 5 mol^−1^ L cm^−1^ [32] or at 515 nm with ε, equal to 4.6 mol^−1^ L cm^−1^ [39]. Precipitation occurred at pH 8.2 giving a blue–green precipitate, due to Co(OH)_2_ formation. In case of the 1:1 stoichiometry of cobalt(II):**9**, absorbance at 250 nm gradually increased with the cobalt(II) complexation, as presented in Figure 10 and Figure 11; this is accompanied by the deprotonation of **CoLH_2_** with the equilibrium: ${CoLH}_{2}⇌CoLH+H$ characterized by *pK*_112_ = 3.18. The molar absorption coefficient increased from 273.9 for **CoLH_2_** to 1158.6 for **CoLH**, which can be explained by the liberation of one proton of one carboxylic group. Then, occurred the deprotonation of **CoLH** with the equilibrium CoLH⇌CoL+H characterized by *pK*_111_ = 4.56 with concomitant formation of **Co_2_LH_−1_** among the equilibrium ${Co}_{2}L{⇌{Co}_{2}}_{{LH}_{-1}}+H$, characterized by a *pK*_21-1_ between pH 3 and 4.3. At 250 nm, the molar absorption coefficient for **CoL** was evaluated at 700.4 and that of **Co_2_LH_−1_** at 309.7 as reported in Table 4. The sum of the two values is near from that of **CoLH**, still in accordance with a liberation of a proton from one carboxylic group.

All other cobalt(II):**9** complexes absorbed with a practically equivalent molar absorption coefficient, at pH beyond 7 with deprotonation of nitrogen groups. It is important to mention that two isosbestic points at 245 nm (*pK*_111_ = 4.54) and 265 nm, (*pK*_11-1_ = 8.74) were observed. Based on the titration results, we propose that the two cobalt(II) complexes concerned are, respectively, **CoLH** and **CoL** (CoL+H⇌CoLH) and **CoL** and **CoLH_−__1_**${(CoL⇌CoLH}_{-1}+H)$, which confirms the presence of only two complexed species in equilibrium. No precipitation of the complex, for the ratio 1:1, was observed at high pH.

In case of the 1.5:1 stoichiometry of cobalt(II):**9** complex, absorbance at 250 nm increased regularly with addition of base as shown in Figure 12 and Figure 13.

At low pH, the absorbance still increased during **CoLH_2_** deprotonation into **CoLH**, **CoLH** into **CoL** and **Co_2_L** into **Co_2_LH_−1_**, where a plateau is attained, before increasing drastically during **Co_2_LH_−1_** deprotonation into **Co_2_LH_−2_**, corresponding certainly to a nitrogen group deprotonation. At pH 8.8, precipitation was observed with the appearance of **Co_2_LH_−2_**. Consequently, it was difficult to calculate molar absorption coefficients of the dinuclear species. Before the pH of precipitation, 1:1 and 2:1 species coexisted as explained before. The dinuclear were preponderant as the ratio cobalt(II):**9** was 1.5:1.

In case of the 2:1 stoichiometry of cobalt(II):**9** complex, absorbance increased drastically at 250 nm, as shown in Figure 14 and Figure 15.

At pH 8.2, precipitation was observed with the appearance of **Co_2_LH_−2_**. At 250 nm, absorbance saturated so results are presented at 290 nm.

#### 2.5.3. Complexation Studies of Cobalt(II) with the Ligand **9** by Isothermal Titration Calorimetry (ITC)

ITC is largely used to measure ligand–macromolecule affinity in chemistry or biology and more recently in different domains such as pharmaceutical nanotechnology or metal complexation [40,41,42]. ITC necessitates the experiments to be undertaken at constant pH to eliminate a pH mismatch between the titrant, the solution contained in the syringe, and the solution contained in the cell. Its use prevents also the generation of additional heat from the water autoprotolysis.

Nevertheless, the resulting thermodynamic parameters correspond to apparent values, since metal–ligand complexation is accompanied by additional phenomena, whether deprotonation or association with the buffer. In this context, we have used ITC to evaluate the relative strength of the complexes that the disaccharide can form with one or two cobalt(II) centers, for a defined pH. Considering the importance of buffer impact on the binding constants and the enthalpy changes in the metallic complex formed [43,44], we have chosen to work with piperazine-*N,N*’-bis(2-ethanesulfonic acid) (*Pipes*) and 2-(*N*-morpholino)ethanesulfonic acid (*Mes*) buffers, which are widely used in biological experiments or calorimetric experiments [45,46,47,48,49,50], and they are known to have low affinities with metals. In case of cobalt(II), in NMe_4_Cl 0.1 N, before conducting our ITC measurements, we also verified its affinities with *Pipes* and *Mes* with experiments of potentiometry. The logarithm values of stability constants between cobalt(II) and the two buffers (*CoPipes* and *CoMes*), were determined to be ca. 2, as shown in Table 5, which is negligible compared to values calculated for the two majority species of the cobalt(II):**9** complexes (log ***β_CoL_*** ≈ 12 and log ***β*_*Co*2*LH*_** ≈ 15–16). These results are in accordance with values taken from the literature, when comparing values mentioned for different counter ions used to adjust ionic strength.

A pH of 6 was chosen to conduct all the ITC measurements, as this value limits the number of species formed in solution. In fact, this pH mainly leads to the formation of **CoL** and **Co_2_LH_−1_** species from disaccharide in the **LH_2_** form, depending on the disaccharide/cobalt(II) molar ratio, as calculated on the basis of potentiometric and spectrophotometric titrations. A pH of 6 also prevents the precipitation of the complex, which occurs at pH values above 8. Direct (injection of cobalt(II) solutions into **9** solutions; experiments **1** to **3**) and reverse titrations (injection of **9** in solution into cobalt(II) solution; experiment **4**) were conducted, before being submitted to global analysis (a single set of thermodynamic parameters to simulate all 4 ITC experiments). Experimental concentrations are compiled in Table 6.

Figure 16 illustrates an example of direct titration conducted in the presence of *Pipes* and *Mes* buffers. In each case, the presence of exothermic and then endothermic signals confirmed the existence of at least two different interactions, justifying the use of a sequential model including 1:1 and 2:1 cobalt(II):**9** complexes to treat experimental data.

Accordingly, the stability constants, *β_ITC_,* and binding enthalpies, *ΔH_ITC_*, of interactions of the disaccharide ligand with cobalt(II) were obtained by fitting ITC isotherms (using a nonlinear least-squared procedure applied simultaneously to experiments **1** to **4**). Then, from the above experimental parameters, the free energy of binding (*ΔG_ITC_*) and entropy change (*ΔS_ITC_*) could be determined from the standard thermodynamic relationship, (*ΔG_ITC_* = −RT ln *K_ITC_* = *ΔH_ITC_* − T*ΔS_ITC_*). Adequation between the theoretical and experimental isotherms obtained is depicted in Figure 17. A very satisfactory theory/experiment agreement was observed for both buffers, confirming that such a sequential 1:1 and 2:1 model can reasonably approximate the complex **9**/cobalt(II)/buffer system at a given pH.

**Scheme 4:**
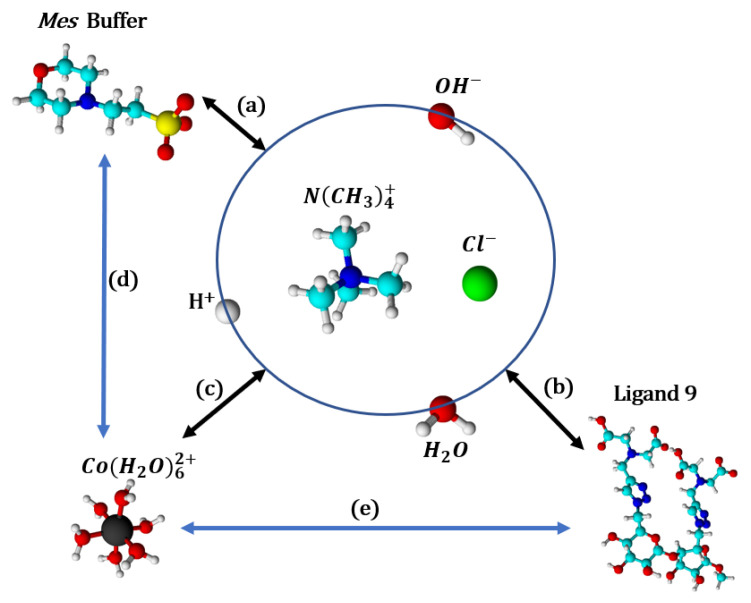
The major interactions, in competition, occurring during an ITC experiment, depending on pH and ionic strength (adjusted with NMe_4_Cl). (a) Buffer and its protonation states; (b) ligand **9** and its protonation states; (c) cobalt(II) and the aqua and hydroxo ligands; (d) cobalt(II) complexation with *Mes* buffer; (e) cobalt(II) complexation with ligand **9.** *Mes* and ligand **9** are on their zwitterionic form as represented in Figure 18. Color of atoms: black: cobalt(II), light blue: carbon, dark blue: nitrogen, green: chloride, red: oxygen, white: hydrogen, yellow: sulfur.

**Scheme 1:**
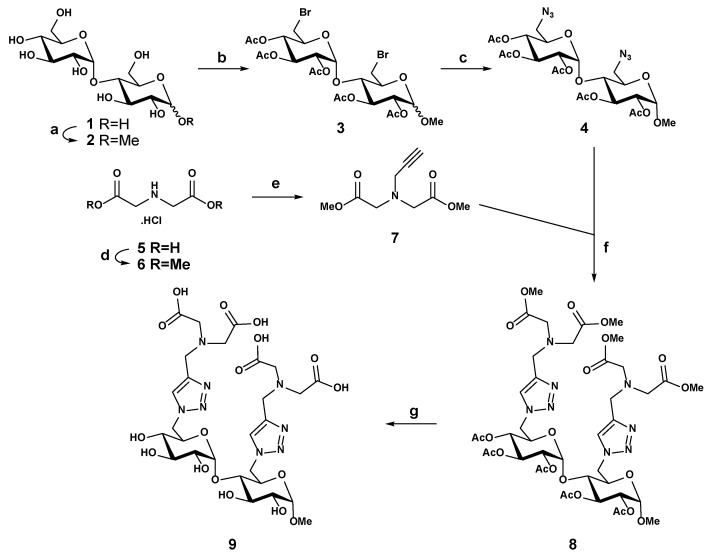
Reagents and conditions: **a**. MeOH/Amberlite 120 (H^+^), reflux, overnight (30%); **b**. PPh_3_/NBS/DMF, 70 °C, overnight, then Ac_2_O/Pyridine, overnight; **c**. NaN_3_/DMF, 80 °C, 18 h (48%, 3 steps); **d**. MeOH/AcCl, 60 °C, overnight (~100%); **e**. propargyl bromide/DIPEA/CH_2_Cl_2_, 40 °C, overnight (65%); **f**. CuI/DIPEA/acetone, overnight (71%); and **g**. NaOMe/MeOH 4 h, then NaOH/H_2_O, reflux, 6 h (92%).

**Scheme 2:**
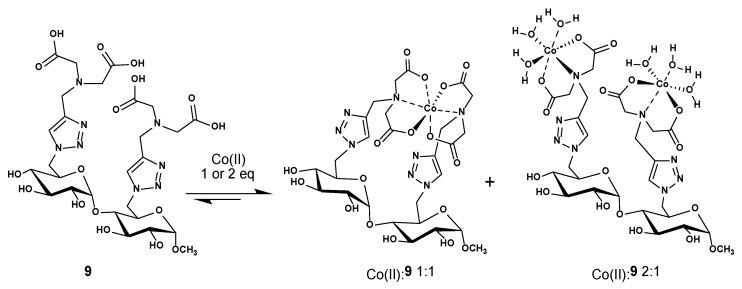
Suggested structures for the cobalt(II):**9** complexes with 1:1 and 2:1 ratio. Charges are omitted for clarity.

**Scheme 3:**
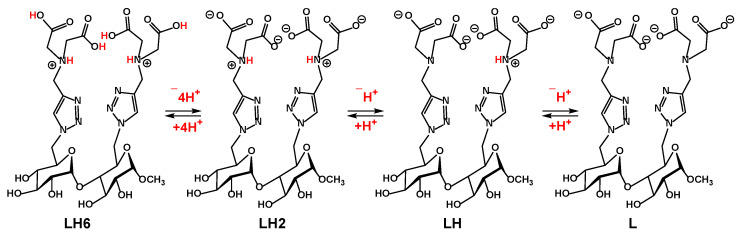
The major protonation states of target disaccharide **9** and their designated notations.

Resulting thermodynamic parameters of interaction of the ligand **9** with the cobalt(II) ions determined by the ITC technique in the *Pipes* and *Mes* buffer solutions with a pH of 6, at 298 K, are summarized in Table 7. It should be stressed again that these values correspond to apparent parameters, since coordination with protons or buffer ions has not been taken into account in the fitting process. Scheme 4 represents the main interactions occurring during an ITC titration experiment with *Mes* buffer, ligand **9**, and cobalt(II) interactions with protons, hydroxide ions, and water (linked together by the autoprotolysis constant), ligand **9** and *Mes* buffer being also each other in competition for cobalt(II) ions.

**Table 7:** Thermodynamic quantities of cobalt(II) binding to ligand **9** in the 70 mM buffer solutions, pH 6, at 298 K, I = 0.1 N (NMe_4_Cl), *pK_w_* = 13.78. Standard deviation in parentheses.

	*Pipes*	*Mes*
Stoichiometry cobalt(II):**9**	1:1 2:1	1:1 2:1
*β_ITC_*/M^−1^	8.4 × 10^5^ (1.10^5^) − 5.1 × 10^9^ (1.10^9^)	1.5 × 10^6^ (6.10^5^) − 9.9 × 10^9^ (6.10^9^)
*ΔH_ITC_*/kcal mol^−1^	−2.79 (0.04) − 1.13 (0.11)	−4.71 (0.09) – −1.02 (0.19)
*TΔS_ITC_*/kcal mol^−1^	5.28 (0.11) − 14.36 (0.23)	3.71 (0.34) − 12.60 (0.61)
*ΔG_ITC_*/kcal mol^−1^	−8.07 (0.7) – −13.23 (0.12)	−8.42 (0.25) – −13.62 (0.42)

The observed constants of complexation for the complex cobalt(II):**9** 1:1 and 2:1 were, respectively, equal to *K_11,obs_*= 8–15 ×10^5^ M^−1^ and *K_21,obs_* = 6–7 × 10^3^ M^−1^, which results in a cumulative observed stability constant equal to *β_21_* = 5–10 × 10^9^ M^−2^. At this step, it is possible to make the hypothesis that a chelate effect could explain the high affinity observed for the 1:1 complex (one cobalt(II) associated with two iminodiacetate groups **9**), which is 10-fold higher than the individual interaction that each cobalt(II) experienced within the 2:1 complex $(\sqrt{\beta_{21}}={7-10\;\times \;10}^{4}M^{-1}$).

**Figure 18:**
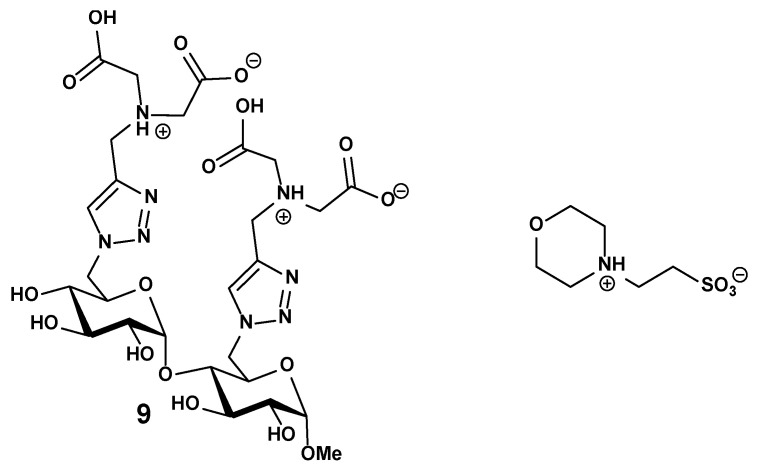
Zwitterionic forms of ligand **9** and *Mes* buffer.

### 2.6. Capture and Release of Cobalt(II)

In order to validate the capacity of the disaccharide ligand **9**, grafted with two iminodiacetates functions, to reduce metal pollution such as cobalt(II), two experiments have been performed. The first one aims to prove the property of ligand **9** to extract cobalt(II) in aqueous solution. The second one was designed to show the release the metal ion and the recycling of the ligand to open the way for a future remediation application. To quantify the cobalt(II), ICP-AES has been selected, as we can effectively titrate the metal ion in trace amounts in medium using this very sensitive technique.

#### 2.6.1. Capture of Cobalt(II)

Ligand **9** was tested with one and two equivalents of cobalt(II) using a 1 g/L solution of CoCl_2_.6H_2_O. The complexation experiments were carried out in triplicates at neutral pH and room temperature during one hour under gentle stirring. The weakly bound cobalt(II) cations were eliminated by dialysis using Biotech Cellulose Ester Dialysis Membrane (Biotech^®^ CE tubing MWCO 100–500 D from spectrum laboratories) and the resulting solutions were lyophilized. ICP-AES measurements were performed on each complex with the experimental ratio as the average on triplicates. We observed for the complex cobalt(II):**9** a ratio of 0.82:1 and 1.60:1 with the ratio 1:1 and 2:1, respectively. The values obtained are below the theoretical results due to a loss of cobalt(II) during the dialysis process (18%).

#### 2.6.2. Release of Cobalt(II)

Desorption experiments were performed to release the cobalt(II) for a recycling strategy and to regenerate the modified disaccharide. This study has been carried out with the ratio cobalt(II):**9** 2:1 in aqueous solution by passing through a column containing a slight excess of acid ion exchange resin (Chelex^®^). Chelex 100 a weak acidic resin, is composed on iminodiacetate acid immobilized on a styrene-divinylbenzene copolymer. Passage of a solution of metal chelate through the resin generates the capture of metal on the support by a competing ion-exchange mechanism [51]. The competition depends on the experimental conditions and the metal binding constant. For instance, IDA ligand (log ***β_CoL_*** ≈ 7) does not compete with the sorption of the metal ion on the resin. In contrast, the metal with EDTA ligand with higher binding constant (log ***β_CoL_*** ≈ 16–18) than IDA is less retained by the resin. A total extraction needs longer time. We aim to prove that the ligand **9** with this intermediate binding constant (log ***β_CoL_*** ≈ 12.3) can both generate stable complex and make possible a quick decomplexation on column of Chelex validating the recyclability of the process. The fractions collected were lyophilized and subsequently analyzed by ICP-AES. The average results of ICP-AES showed a decrease in the ratio from 1.60:1 to 0.30:1. A partial decomplexation (81%) occurred and we recovered 1.3 equivalents of cobalt(II) after only one run of filtration. After decomplexation with Chelex^®^, the ESI-TOF HRMS spectrum revealed mainly the presence of uncomplexed ligand **9** with the peaks at *m*/*z* 365.1060 [M + Na]^+^ and *m*/*z* 373.1177 [M-2H]^2−^, respectively. Nonetheless, the presence of mononuclear 1:1 cobalt(II):**9** complex was observed with *m*/*z* 401.5765 [ligand **9**-4H + Co]^2−^. These results highlight the stronger chelating effect of the two IDA groups with the metal ion, as more resin is needed to remove quantitatively the cobalt(II). Nevertheless, it is possible to displace the complexation equilibrium with simple filtration on cation exchange resin to recover the cobalt(II) for a new application and to regenerate the active ligand **9** for another chelation cycle. As application, the immobilization of the ligand **9** on magnetic insoluble solid support is in progress.

## 3. Materials and Methods

### 3.1. NMR Study

NMR samples were prepared as follows: (a) 5.8 mg of 1:1 Co:**9** complex were dissolved in 0.6 mL of D_2_O; (b) 17.8 mg 2:1 Co:**9** complex were dissolved in 0.6 mL of D_2_O. ^1^H and ^13^C NMR spectra of 1:1 and 2:1 cobalt(II):**9** complexes were recorded using a Bruker Avance III HD 400 NMR spectrometer equipped with a 5 mm BBO {^1^H, X} probe and a z-gradient unit. NMR spectra were recorded at 298 K in D_2_O. Chemical shifts (δ) were reported in ppm (relative to TMS). The residual solvent signal was used as reference (for D_2_O: δ_HDO_ = 4.79 ppm) and the chemical shifts were converted to the TMS scale because of the paramagnetic effect of cobalt(II), NMR experiments had to be specially adjusted.

### 3.2. Ligand ***9*** and Buffers Protonation Studies by Potentiometry

All solutions were prepared using milli-Q water and degassed by bubbling a stream of argon through the solution in order to remove dissolved CO_2_ and to prevent cobalt(II) oxidation. Their ionic strength was adjusted to 0.1 N with NMe_4_Cl. The 0.01 M HCl solution was prepared from standard Titrisol (Merck, Darmstadt, Germany) and adjusted to I = 0.1 M with NMe_4_Cl. This solution was used to calibrate the Metrohm combined glass microelectrode. Carbonate-free NMe_4_OH solution (ca. 0.05 M) was standardized against potassium hydrogen phthalate (RPE, Carlo Erba, Milan, Italy) by potentiometry, recording e.m.f. (mV) after each base addition. The ionic product of water was determined by titrating an acetic solution in 0.1 M NMe_4_Cl at different concentrations. Under these conditions, *pK_w_* = 13.78. This value was used in the calculations. Automatic titrations were carried out using a 905 Metrohm Titrando apparatus equipped with a 800 Dosino 2 mL burette; the system is connected to a computer. The delivery of titrant, data acquisition, monitoring for e.m.f. stability were controlled by the Tiamo 2.2 software. Injection volumes value were 4 µL. Sample solutions were titrated in a double-walled glass vessel, fitted with a sealed lid containing ports for the glass combination electrode (Metrohm #6.0234.100), Teflon anti-diffusion burette tip and Teflon gas line. All equilibrium measurements were carried out in 4.0 mL sample volumes under magnetic stirring, at 25 ± 0.2°C, in the thermostated cell using a thermocirculator bath (Julabo HE cryostat), under an argon stream. The ligand stock solution was prepared by dissolving a weighted amount of ligand in an appropriate amount of 0.1 M NMe_4_Cl solution. In our experiments, a stock solution of the ligand **9** (10^−3^ M) was used. Titrations with NMe_4_OH were carried out in the presence of extra-HCl to ensure initial protonation of carboxylate groups. The initial volume of the measured solution was 4.0 mL and the ligand concentration was fixed at 5.0 × 10^−4^ M. Error in titer has been fixed to 0.01 mL and in electrode reading to 0.01 mV. Before the first point acquisition, the reaction cell was sealed, and the solution was stirred and sparged with argon for 10 min. Finally, the sample solution was titrated to ca. pH 12 with standardized NMe_4_OH (ca. 0.05 M) in a monotonic mode. Increments of base added were fixed at 4.0 µL. The electrode was monitored for up to 6 min or until its drift was less than 0.4 mV/min, before recording a mV reading. Cobalt(II) did not oxidized during experiments into cobalt(III), thanks to argon bubbling before experiments. So, cobalt(III) did not disturb equilibria which, otherwise, could have taken days or weeks if this latter had formed, with the drift value chosen. The protonation constants of the ligand **9** were determined from ten titrations to reduce experimental errors. For buffers, piperazine-*N,N’*-bis(2-ethanesulfonic acid) (*Pipes*, MW = 302.4 g.mol^−1^) and 2-(*N*-morpholino)ethanesulfonic acid (*Mes*, MW = 195.2 g.mol^−1^), were used to maintain the pH constant to 6 in ITC experiences. Protonation constants were determined by titrating a solution of either *Pipes* (21.45 mM) or *Mes* (72,8 mM) into a solution of NMe_4_Cl (0.1 N). For *Pipes* buffer, the initial volume of the measured solution was 4.0 mL and ligand concentration was between 2.4 and 5.4 × 10^−3^ M with extra-HCl, so that [HCl]/[*Pipes*] = 0 to 1. For *Mes* buffer, the initial volume of the measured solution was 4.0 mL and ligand concentration was between 1.11 and 1.15 × 10^−2^ M with extra-HCl, so that [HCl]/[*Mes*] = 0.21. Each sample solution was titrated with a standardized NMe_4_OH solution (ca. 0.05 M), as previously described for the disaccharide ligand. Concentration of the ligand species was calculated from potentiometric data (e.m.f.) during the determination of the protonation constants, and the data were processed with the general computation program HYPERQUAD [30] to refine the stability constants, *β*.

The model has been specified by defining a set of equilibrium constants from Equations (1)–(9). In HYPERQUAD, the stability constants, *β*, (not log *β*) are the parameters that are refined and by default, a standard deviation is labelled as excessive if it is more than 33% of the parameter value. Although this value was user definable, the default value of 33% was used for this work.

### 3.3. Stability Constant Measurement Between Cobalt(II) and Ligand ***9*** or Cobalt and Buffers by Potentiometry

Stock solutions of cobalt(II) chloride were prepared from commercially available cobalt(II) chloride hexahydrate (Alfa Aesar, HaverHill, MA, USA) dissolved in distilled water, and subsequently diluted to the required concentrations. Ionic strength of the solution was adjusted to 0.1 M with NMe_4_Cl. The exact concentration of metal chloride stock solution was determined by ICP-MS. Stability constants of cobalt(II)-disaccharide ligand **9** were determined from fourteen titrations, using solutions containing 0 to over 2.355 equivalents of cobalt (II) chloride. The initial ligand and HCl concentrations were 5.3 × 10^−4^ M and 3.65 × 10^−3^ M, respectively. Stability constants of cobalt(II)-*Pipes* were determined from solutions containing 0 to 0.23 equivalent of cobalt(II) chloride; the initial ligand and HCl concentrations were 8 × 10^−3^–1.5 × 10^−2^ M and 2.5 × 10^−3^ M, respectively. Stability constants of cobalt(II)-*Mes* were determined from solutions containing 0 to 0.15 equivalent of cobalt(II) chloride; the initial ligand and HCl concentrations were 1.11 × 10^−2^ M and 2.5 × 10^−3^ M, respectively. The same method was used to determine the metal–ligand stability constants. The computer program HySS was used to obtain the speciation distributions curves [31].

### 3.4. Ligand ***9*** Protonation Studies by Spectrophotometry

For the preparation of the solution, the readers are referred to the paragraph concerning potentiometric experiments. In the spectrophotometric experiments, the ligand **9** concentration of the stock solution was 9.5 × 10^−3^ M. The initial volume of the measured solution was 4.0 mL, ligand concentration was fixed at 2.37 × 10^−3^ M and extra-HCl at 1.55 × 10^−2^ M. Before the first point acquisition, the reaction cell was sealed, solution was stirred and sparged with argon for 10 min. Finally, the sample solution was titrated to ca. pH 12 with standardized (ca. 0.1 N) NMe_4_OH. Increments of base, added manually with the burette from the potentiometer, were varied between 25 and 50 µL. Once an equilibrium was reached after the addition of base, the potential reading was recorded using a glass electrode from the titrator and a scan, with a Cary UV–Visible Varian spectrophotometer equipped with a dip probe, was launched to measure absorbance at every wavelength between 190 and 900 nm. The blank was 0.1 M NMe_4_Cl. The protonation constants of the ligand **9** were evaluated with Hypspec program from the HYPERQUAD suite and compared to those obtained with potentiometric method.

### 3.5. Stability Constant Measurement Between Cobalt(II) and Ligand ***9*** by Spectrophotometry

Stock solutions of cobalt(II) chloride were prepared as described in the potentiometric method section. Stability constants of cobalt(II):**9** with the stoichiometries 1:1, 1.5:1 and 2:1, were determined from manual titrations described above; the initial ligand and HCl concentrations were 2.27 × 10^−3^ M and 1.55 × 10^−2^ M, respectively.

### 3.6. Thermodynamic Parameters of Cobalt(II) Binding to Ligand ***9*** by Isothermal Titration Calorimetry

Isothermal Titration Calorimetry (ITC) experiments were performed at 298 K using a MicroCal PEAQ-ITC (Malvern Panalytical, Malvern, UK) instrument equipped with a 200 µL adiabatic- sample cell, a reference cell (filled with milli-Q water in this case) and a 40 µL syringe, and the operation was piloted by the MicroCal PEAQ-ITC Control software. The differential power required between the reference and sample cells were measured in order to maintain a zero balance in temperature between the two cells. The heat normalized per mole on injectant were processed with MicroCal PEAQ-ITC Analysis software version 1.21. The calibration of the MicroCal PEAQ-ITC calorimeter was carried out using electrically generated heat pulses. The CaCl_2_–EDTA titration was performed to check the apparatus and the results (*n*, *K*, *ΔH*) were compared with those obtained for the same samples (test kit) from MicroCal. All solutions were degassed using a Thermovac instrument (Malvern Panalytical, Malvern, UK) before the titrations were performed. Titrant was injected at 150 s intervals to ensure that the titration peak returned to the baseline prior the next injection. Each injection lasted 6 s. To achieve a homogeneous mixing in the cell, the stirrer speed was kept constant at 750 rpm. An initial 0.4 µL injection sample was discarded from each data set to remove the effect of titrant diffusion across the syringe tip during the equilibration process. The experiment consisted of 19 injections (2 µL each, except for the first injection, where only 0.4 µL was injected) of ca. 1–15 mM solution of appropriate metal salt into the reaction cell, initially containing buffered solution of ca. 0.2–1 mM disaccharide ligand **9** (I = 0.1 M NMe_4_Cl). For each titration of cobalt(II) chloride/disaccharide **9**, a background titration was performed using identical titrant with the buffer solution (70 mM, pH 6) placed in the sample cell. The buffers used in this study were *Pipes* (piperazine-*N,N’*-bis(2-ethanesulfonic acid))) and *Mes* 2-(*N*-morpholino)ethanesulfonic acid) (Sigma Aldrich GmbH, ST Gallen, Swiss). Inverse titrations were also conducted when disaccharide ligand **9** was injected into the reaction cell that contains a solution of cobalt(II) chloride.

### 3.7. Complexes Co(II):***9*** and Samples Preparation and ICP-AES Study

The 1:1 and 2:1 cobalt(II):**9** complexes were prepared in triplicate using a stock solution of 1 g/L of cobalt(II). The ligand **9** was stored under a desiccation bell for 2 days before use. After adding 1 or 2 equivalents of CoCl_2_.6H_2_O to a solution of **9**, the pH was adjusted to 7 with a 0.1M HCl solution. The reaction was stirred for 1 h at room temperature. The crude mixture was dialyzed to remove the free cobalt ions.

Dialyses were performed using a Biotech Cellulose Ester Dialysis Membrane: Biotech^®^ CE tubing MWCO 100–500 D, Nominal Flat Width: 31 mm, Diameter: 20 mm, Vol/Length: 3.1 mL/cm, Length 10 m/33 ft from Thermo Fisher Scientific, Waltham, MA, USA. Cobalt(II) release was carried out by filtering through a column containing Chelex^®^ 100 (sodium form), which is crosslinked polystyrene-iminodiacetate ion exchange resin, 100–200 mesh particle size, binding capacity 0.6 mg_eq_/g). ICP-AES measurements were performed on an iCAP 6000 series (Thermo Fisher Scientific, Waltham, MA, USA) using the axial view. The quantification of cobalt(II) was performed at 228.61 nm. Both standard solutions of cobalt(II) and the samples were prepared in 2% HNO_3_. The dialyzed solution was lyophilized and stored under desiccation bell. For ICP-AES measurements, approximately 1 mg of sample was weighted using a high precision balance, and then dissolved in 1 mL of milli-Q water. The solution was further diluted to 10 or 20 fold with milli-Q water containing 2% (*v*/*v*) HNO_3_, according to the expected amount of cobalt(II). All the measurements were repeated at least three times.

### 3.8. Formation of Complexes Cobalt(II)^+^:***9*** and Analysis

#### 3.8.1. Formation of Complex Co:**9** 1:1 and Analysis

Methyl 6,6′-dideoxy-6,6′-di-*N*-(4-((bis(carboxymethyl)amino)methyl)-1*H*-1,2,3-triazol-1-yl)-α-D-glucopyranosyl-(1→4)-α-D-glucopyranoside, disodium salt **9** (2.23 mg, 3.0 µmol, 1 eq.) was dissolved in ultrapure water (8.0 mL) and the pH of the solution was adjusted to 7 with a 0.1M sodium hydroxide solution. Then, cobalt(II) chloride hexahydrate (0.18 mg, 3.0 µmol, 1 eq.) was added to the reaction mixture and the pH was adjusted to 7 with a 0.1M hydrochloride solution. The reaction was left stirring at room temperature for 1 h. The mixture was then transferred into a dialysis tube to remove the excess of cobalt(II). After changing water five times over a day, the mixture in the dialysis bag was collected and lyophilized. An amount of 2.35 mg (97.5% of yield) of the desired complex cobalt(II):**9** was obtained as a pink solid. The formed cobalt(II) complex was determined by ICP-AES measurements. TOF HRMS ESI-MS: *m*/*z* calc. for C_27_H_37_N_8_O_17_Co [M-H^+^+Co^2+^]^−^ 804.1609, found 804.1613.

#### 3.8.2. Formation of Complex Co:**9** 2:1 and Analysis

Methyl 6,6-dideoxy-6,6-di-N-(4-((bis(carboxymethyl)amino)methyl)-1H-1,2,3-triazol-1-yl)-α-D-glucopyranosyl-(1→4)-α-D-glucopyranoside, disodium salt **9** (21.63 mg, 28.9 µmol, 1 eq.) was dissolved in ultrapure water (8.0 mL) and the pH of the solution was adjusted to 7 with a 0.1M sodium hydroxide solution. Cobalt(II) chloride hexahydrate (3.40 mg, 57.8 µmol, 2 eq.) was added in the reaction mixture and the pH of the mixture was adjusted to 7 with a 0.1M hydrochloride solution. The reaction was left stirring at room temperature for 1 h. The mixture was then transferred into dialysis tube to remove the excess of cobalt(II). After changing water five times over a day, the mixture in the dialysis bag was collected and lyophilized. An amount of 23.24 mg (94.9 of yield) of the desired complex cobalt(II):**9** was obtained as a purple solid. The complexed cobalt(II) amount was determined by ICP-AES measurements. TOF HRMS ESI-MS: *m*/*z* calc. for C_27_H_35_N_8_O_17_Co_2_ [M-H^+^+2Co^2+^]^−^ 861.0784, found 861.0799.

### 3.9. Desorption Experiments

The desorption tests were performed using an ion exchange column packed with Chelex^®^ resin from Sigma Aldrich GmbH, ST Gallen, Swiss. For cobalt(II):**9** with a ratio 2:1 complex, the amount of Chelex^®^ required for the experiment was calculated according to the binding capacity provided by the supplier (0.6 mg_eq_/g) and, the amount of captured cobalt(II) was estimated. Based on the calculation, an excess of 20% excess of Chelex^®^ was used. The Chelex^®^ resin was washed with 500 mL of milli-Q water before use. The complex was then deposited and eluted with 400 mL of milli-Q water.

## 4. Conclusions

We reported the synthesis of a new ligand **9** composed of two iminodiacetate (IDA) functionalities immobilized on a biodegradable disaccharide scaffold. Compound **9** was synthesized in five steps using a “click” reaction as the key step between the 6,6′-diazide of methyl α-maltoside and *N*-propargyl iminodiacetic ester. We studied the capacity of this new ligand to chelate and release cobalt(II) as a model study. Recovery of the valuable cobalt(II) metal using greener ligand is still challenging. An original cobalt(II) NMR experiment was reported in this work, with the two complexes showing pronounced differences in the expanded regions of the spectra, proving the formation of two types of complexes between cobalt(II) and ligand **9** (1:1 and 2:1). Thanks to potentiometry, spectrophotometry, and ITC studies, we proved the formation of the two cobalt(II) complexes with ligand **9**; this improved our knowledge on binding interactions involved during these complexation processes. The binding constants for the 1:1 and 2:1 ratios of complex with ligand **9** were, respectively, determined to be *K*_11,*obs*_ = 8–15 × 10^5^ M^−1^ and *K*_21,*obs*_ = 6–7 × 10^3^ M^−1^, which correspond to a cumulative observed stability constant of *β*_21_ = 5–10 × 10^9^ M^−2^. Consequently, the chelating effect could effectively explain the high affinity observed for the 1:1 complex (one cobalt(II) associated with two iminodiacetate groups), which is 10-fold higher than the 2:1 complex, where each of the two IDA groups interacts alone with a cobalt(II). Taking into account the log *β**_CoL_*** value obtained for the ligand **9** (~11–12) with the stoichiometry 1:1, the strength of the complexation with cobalt(II) can be classified in the following orders: IDA < MIDA < NTA < **9** < EDTA < TTHA < DTPA. Consequently, this new α-maltoside-based ligand **9** has an intermediate complexation ability in comparison to other usual ligands, suggesting that ligand **9** has potential to be used as a remediation agent. To demonstrate this, we further completed a remediation test with water contaminated with cobalt(II). We demonstrated that recovering of chelated cobalt(II) metal was possible using competitive ion exchange treatment with commercially available Chelex^®^ resin, which allowed a recycling of the synthetic ligand for future recovering experiments. This fundamental study using environmentally friendly carbohydrate chelators will open ways to new applications in the remediation and extraction of other metals, providing an effective tool and viable strategy in the new circular economy.

## Figures and Tables

**Figure 1:**
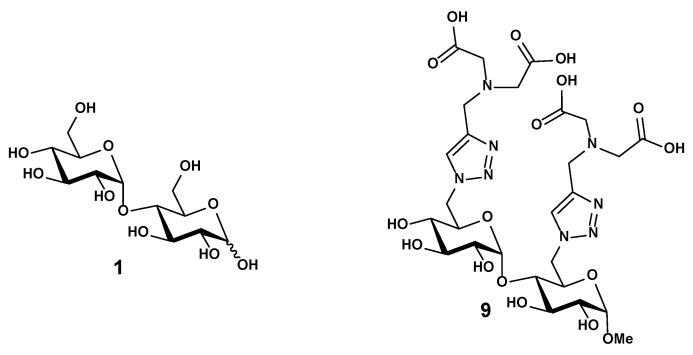
Structure of the maltose **1** and the disaccharide **9** functionalized with iminodiacetate (IDA) ligands.

**Figure 2:**
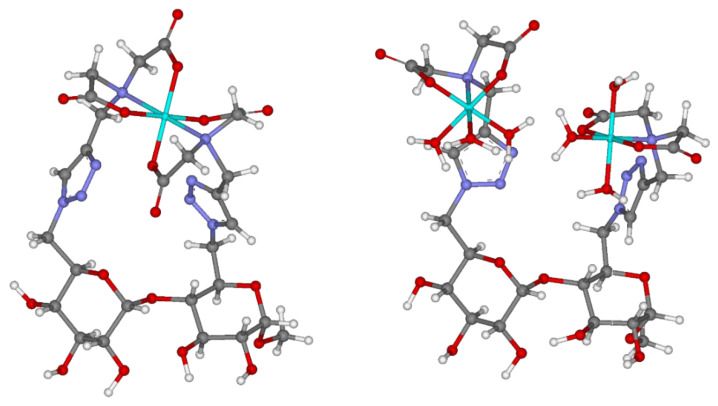
Proposed molecular modeling to illustrate 1:1 and 2:1 cobalt(II):**9** complexes. Color of atoms: light blue: cobalt(II), dark blue: nitrogen, grey: carbon, red: oxygen, white: hydrogen.

**Figure 3:**
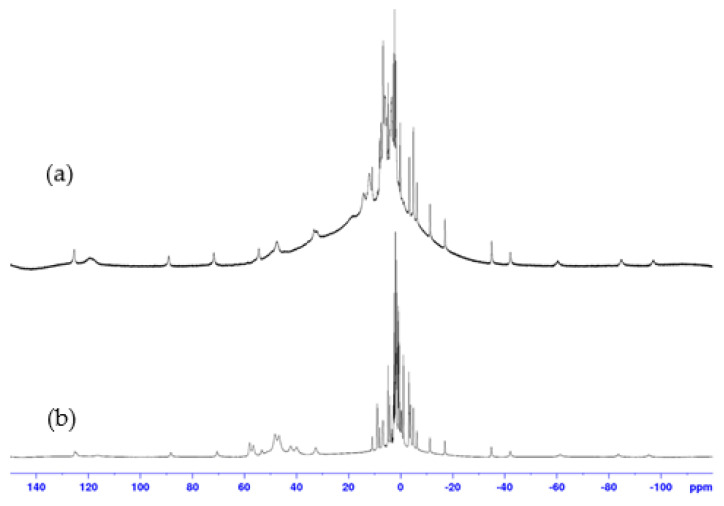
^1^H NMR spectra (400 MHz, D_2_O, 298 K) of: (**a**) 1:1 and (**b**) 2:1, cobalt(II):**9** complexes.

**Figure 4:**
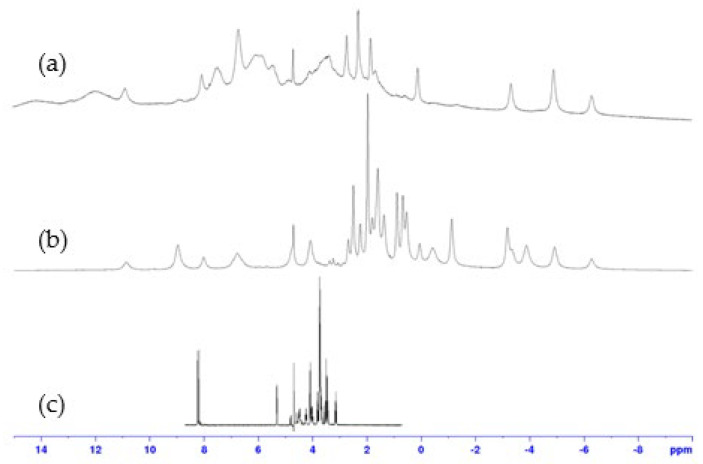
Expansion of ^1^H NMR spectra (400 MHz, D_2_O, 298 K) of: (**a**) 1:1, (**b**) 2:1, cobalt(II):**9** complexes, compared to (**c**) ligand **9**.

**Figure 5:**
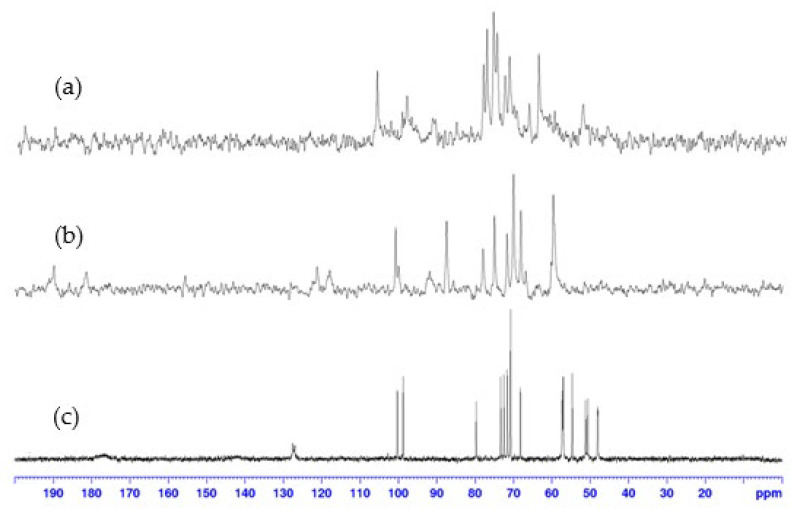
^13^C NMR spectra (100 MHz, D_2_O, 298 K) of: (**a**) 1:1, (**b**) 2:1, cobalt(II):**9** complex, compared to (**c**) ligand **9**.

**Figure 6:**
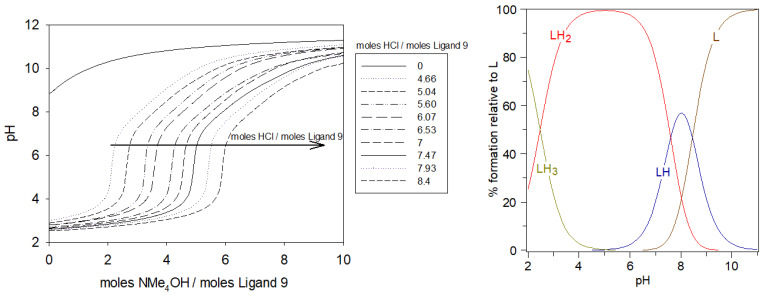
Potentiometric titrations of the ligand **9** (**left**). Ligand **9**: 2.12 μmol; using extra-HCl: 0–17.8 μmol in 0.1 M NMe_4_Cl; total initial volume: 4 mL; burette: [NMe_4_OH] = 0.05 M; [ligand **9**]: 5.0 × 10^−4^ M. Calculated distributions curves (**right**) of different protonated species of **9** at different pH.

**Figure 7:**
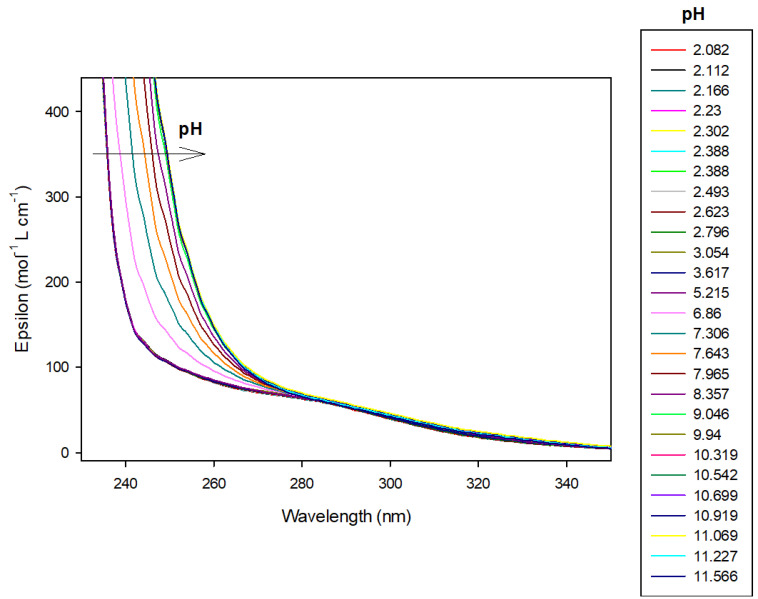
Spectrophotometric titrations of the ligand **9** alone in the absence of cobalt(II). Ligand **9**: 9.5 µmol; extra HCl: 62 µmol in 0.1 M NMe_4_Cl; total initial volume: 4.0 mL. Burette: [NMe_4_OH] = 0.1 M. Blank: 0.1 M NMe_4_Cl.

**Figure 8:**
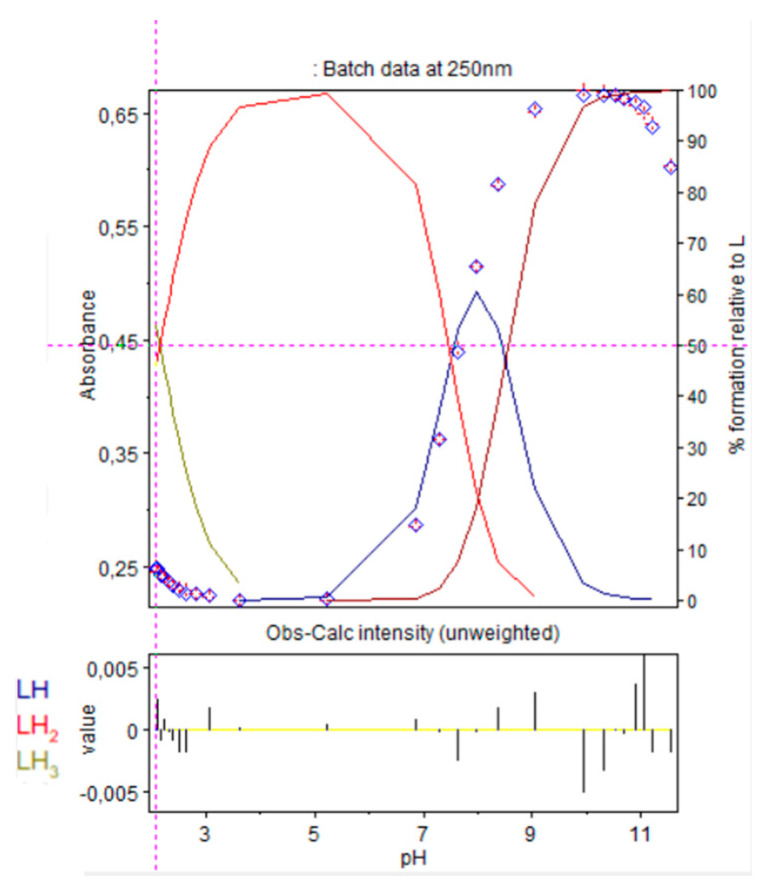
Refinement of the spectrophotometric scans from selected wavelengths: 238 to 298 nm. Ligand **9** concentration from 2.375 mM at pH 2.08 to 1.81 mM at pH 11.56. Presentation of the results for absorption wavelength 250 nm from the Hypspec program. Diamond shape: experimental values, solid curves: calculated speciation.

**Figure 9:**
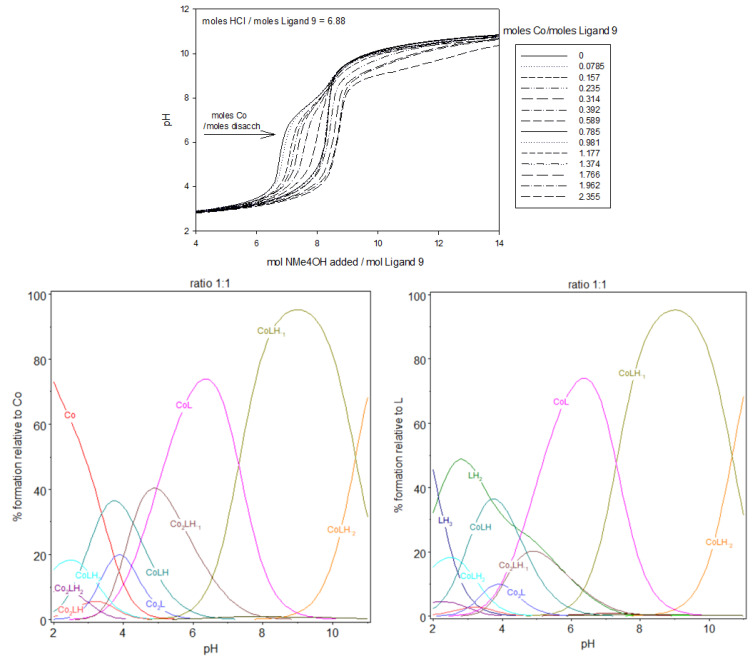

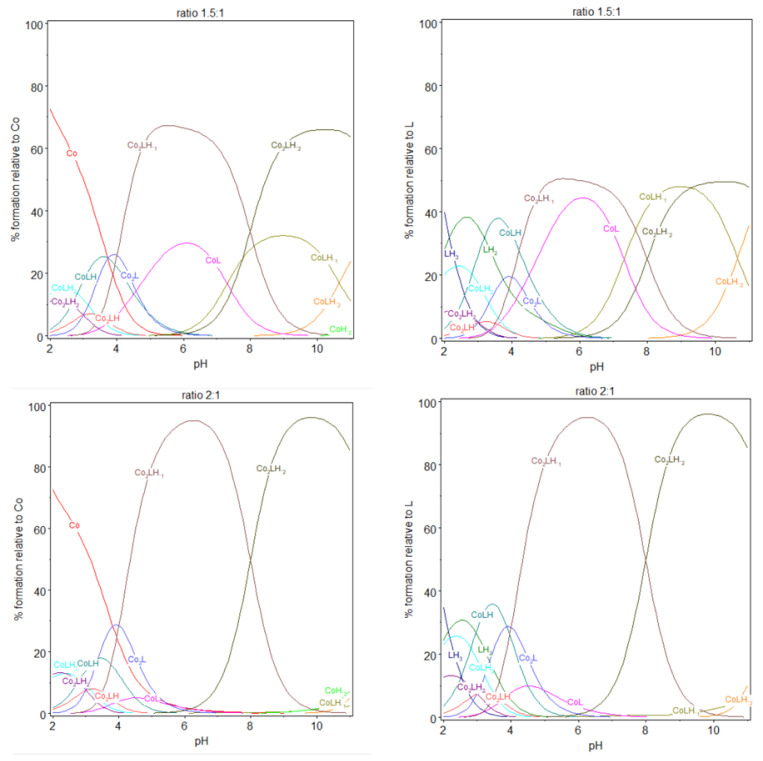
Potentiometric titrations of the ligand **9** in the presence of cobalt(II). Fourteen titrations were performed. Ligand **9**: 2.12 µmol; extra HCl: 14.57 µmol in NMe_4_Cl (0.1 M); total initial volume: 4.0 mL. Burette: [NMe_4_OH] = 0.05 M. And the calculated distribution curved of 1:1, 1,5:1, and 2:1 complex, relative to L or cobalt(II), during titration. [9] = 5.0 × 10^−4^ M.

**Figure 10:**
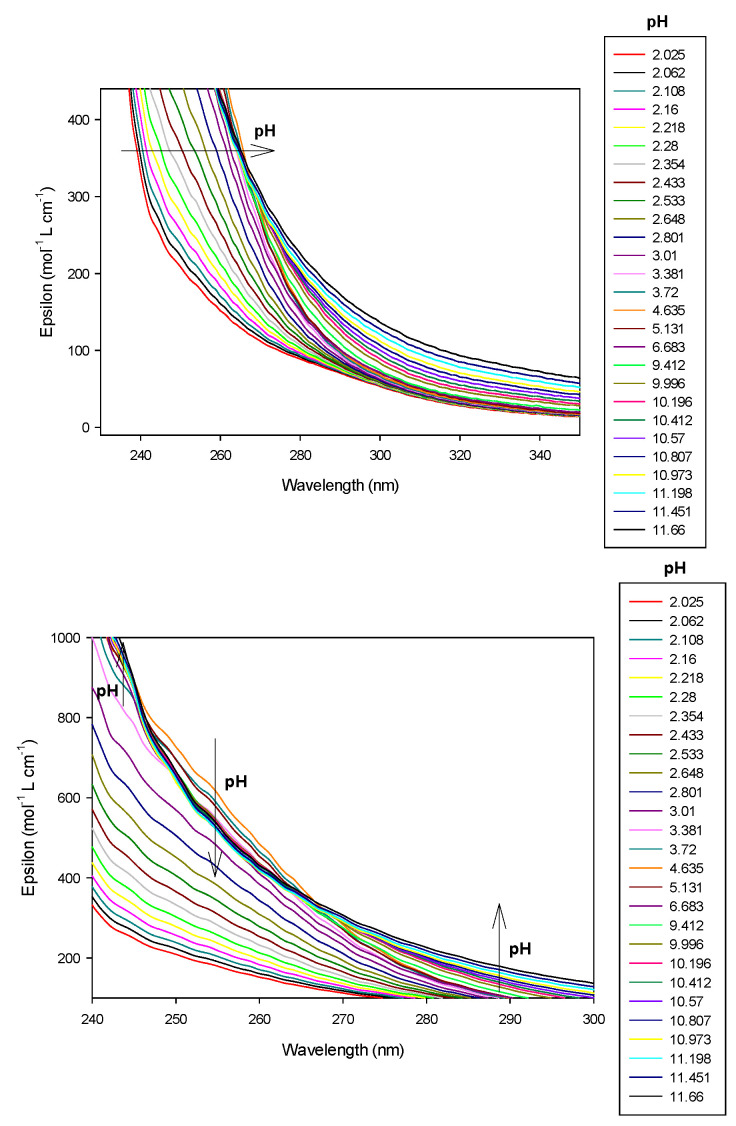
Spectrophotometric titrations of the ligand **9** in the presence of cobalt(II). Ratio cobalt(II):**9**: 1:1. Ligand **9**: 9.5 µmol; cobalt(II): 9.5 µmol; extra HCl: 62 µmol in 0.1 M NMe_4_Cl; total initial volume: 4.0 mL. Burette: [NMe_4_OH] = 0.1 M. Blank: 0.1 M NMe_4_Cl. **Top** and **Bottom**: isosbestic points at 245 nm and 265 nm. Arrows indicate increasing pH.

**Figure 11:**
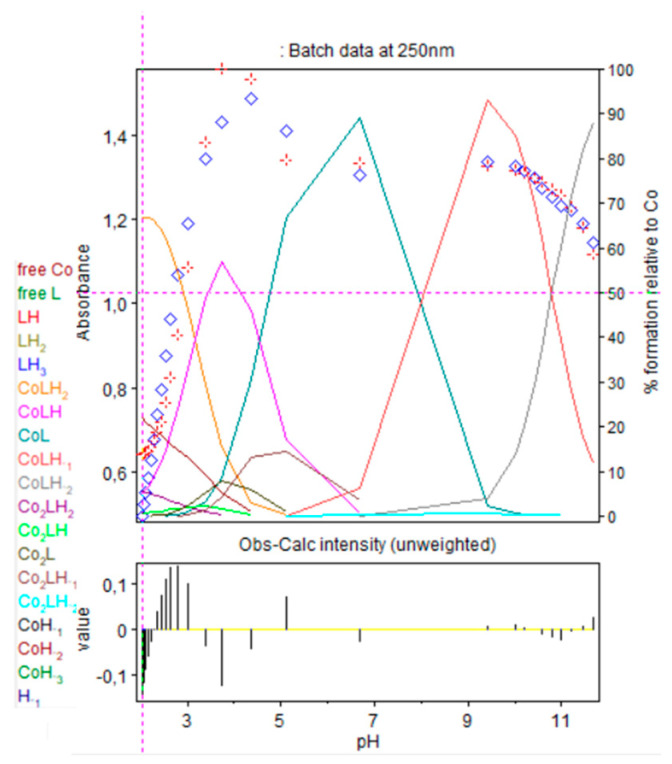
Refinement of the spectrophotometric scans for the 1:1 cobalt(II):**9** complex from selected wavelengths: 240 to 340 nm. Ligand **9** concentration from 2.375 mM at pH 2.02 to 1.719 mM at pH 11.66. Presentation of the results for absorption wavelengths 250 from the Hypspec program. Diamond shape: experimental values, solid curves: calculated speciation. Calculated distribution curves were similar to those obtained by the potentiometric method, as shown in Figure 9.

**Figure 12:**
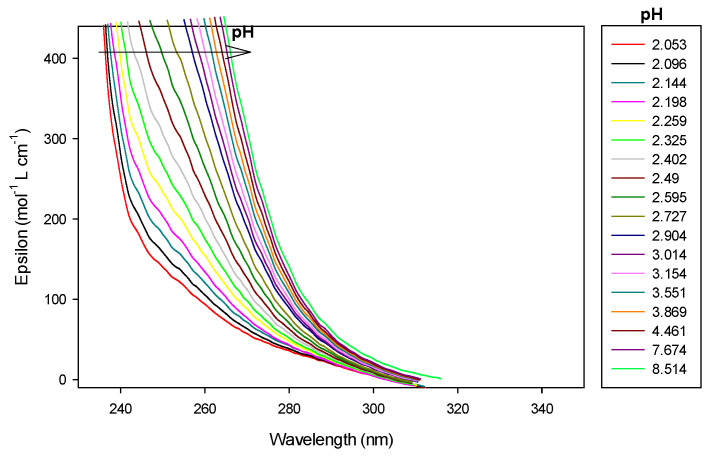
Spectrophotometric titrations of the ligand **9** in the presence of cobalt(II). Ratio cobalt(II):**9**: 1.5:1. **9**: 9.5 µmol; cobalt(II): 14.25 µmol; extra HCl: 62 µmol in 0.1 M NMe_4_Cl; total initial volume: 4.0 mL. Burette: [NMe_4_OH] = 0.1 M. Blank: 0.1 M NMe_4_Cl. Arrows indicate increasing pH.

**Figure 13:**
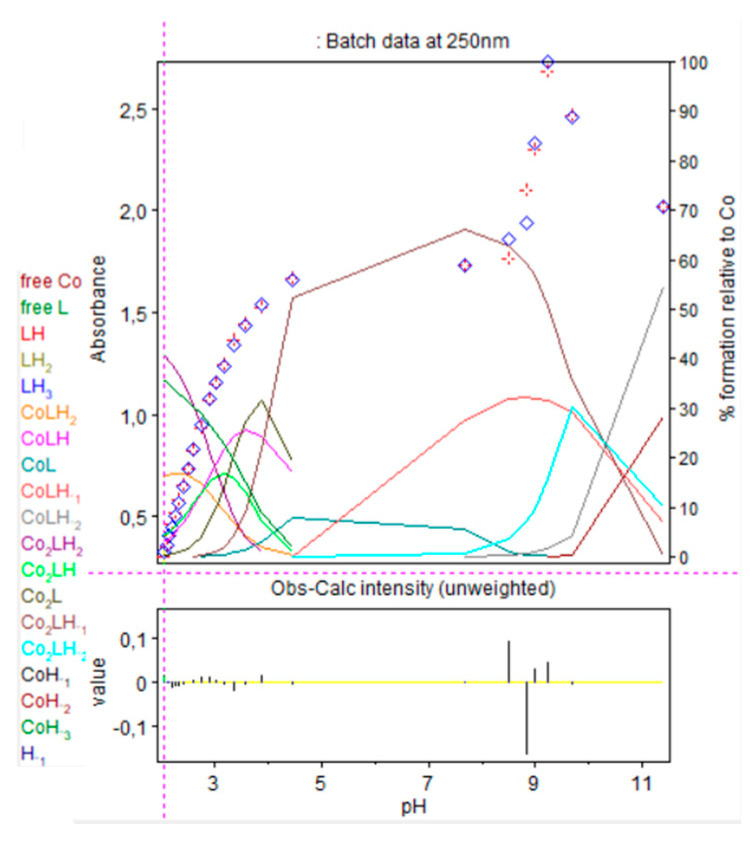
Refinement of the spectrophotometric scans for the 1.5:1 cobalt(II):**9** ratio from selected wavelengths: 240 to 299 nm. Ligand **9** concentration from 2.323 mM at pH 2.05 to 1.755 mM at pH 11.40. Presentation of the results for absorption wavelengths 250 nm from the Hypspec program. Diamond shape: experimental values, solid curves: calculated speciation. Calculated distribution curves were similar to those obtained by the potentiometric method as shown in Figure 9.

**Figure 14:**
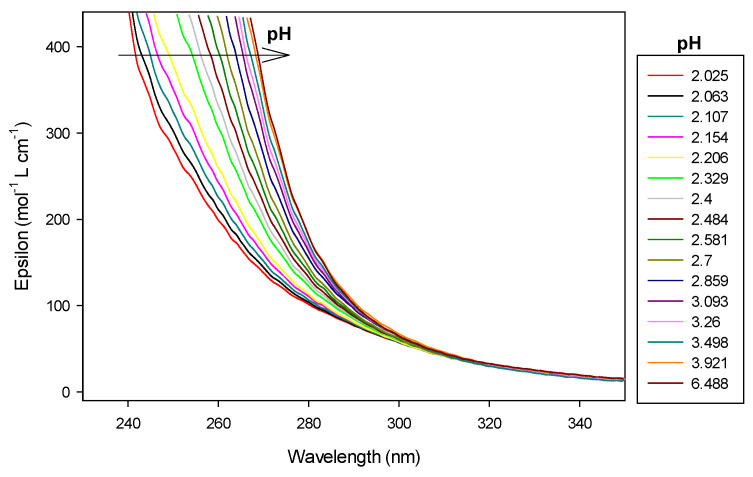
Spectrophotometric titrations of the ligand **9** in the presence of cobalt(II). Ratio cobalt(II):**9**: 2:1. **9**: 9.5 µmol; cobalt(II): 19 µmol; extra HCl: 62 µmol in 0.1 M NMe_4_Cl; total initial volume: 4.0 mL. Burette: [NMe_4_OH] = 0.1 M. Blank: 0.1 M NMe_4_Cl. Arrows indicate increasing pH.

**Figure 15:**
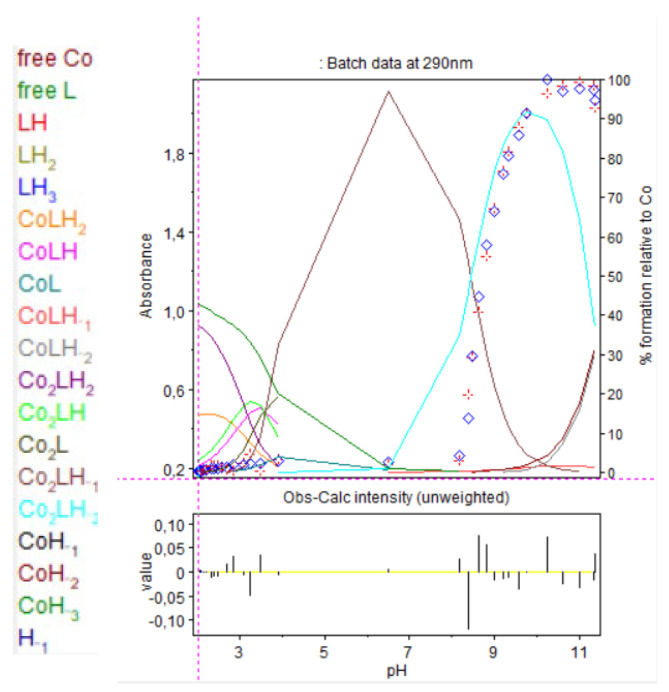
Refinement of the spectrophotometric scans for the 2:1 cobalt(II):**9** complex from selected wavelengths: 260 to 320 nm. Ligand **9** concentration from 2.375 mM at pH 2.02 to 1.681 mM at pH 11.35. Presentation of the results for absorption wavelength 290 nm from the Hyperspec program. Diamond shape: experimental values, solid curves: calculated speciation. Calculated distribution curves were similar to those obtained by the potentiometric method as shown in Figure 9.

**Figure 16:**
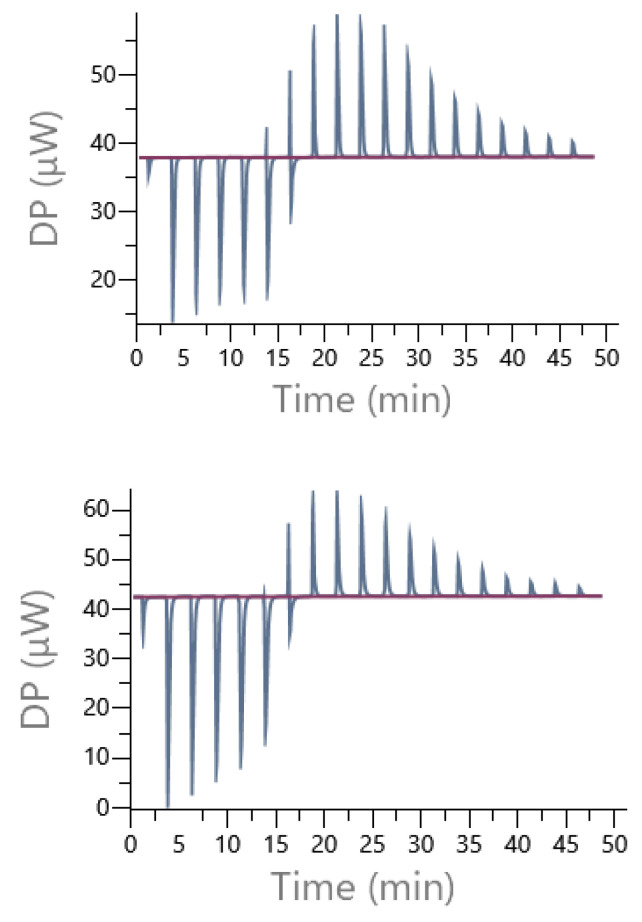
Example of the raw DP (µW) obtained from isothermal titrations of the direct cobalt(II) (14 mM) injection into the cell containing 1 mM of ligand **9** in presence of *Pipes* buffer (**top**) or *Mes* 70 mM, pH 6 (I = 0.1 N NMe_4_Cl) (**bottom**).

**Figure 17:**
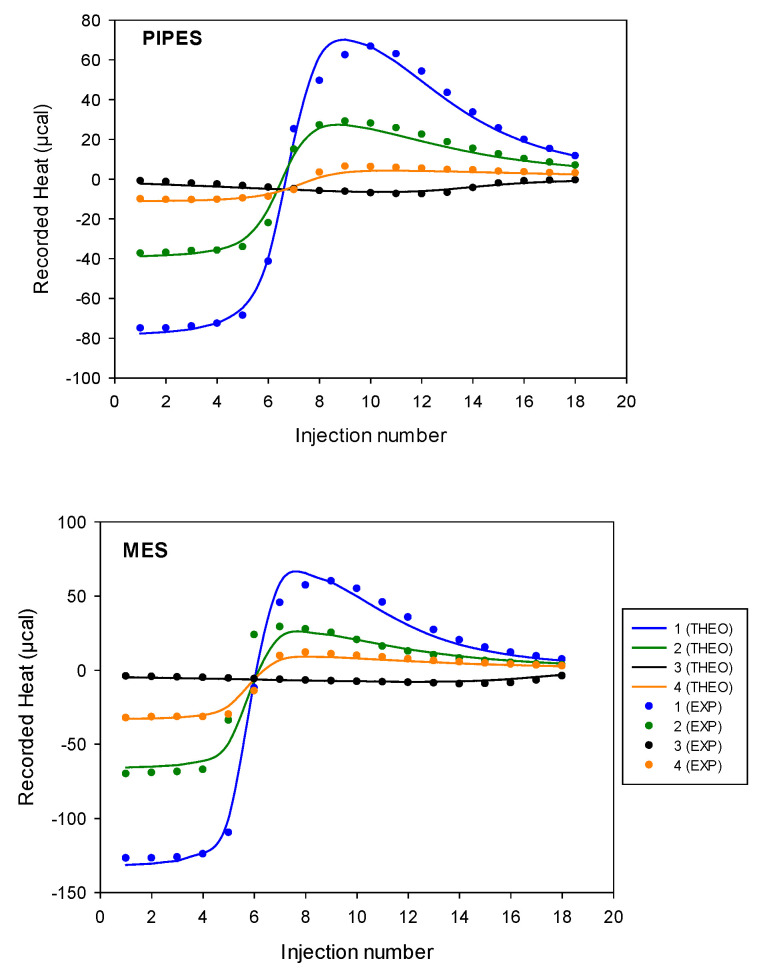
Heat (µcal) calculated from isothermal titrations of the direct cobalt(II) injection into the cell containing **9** or inverse titrations of **9** into the cell containing cobalt(II) in presence of *Pipes* or *Mes* buffer 70 mM, pH 6 (I = 0.1 N NMe_4_Cl). Nonlinear least-squares model used: sequential binding sites. Concentrations values for experiments **1** to **4** are given in Table 6.

**Table 1:** Logarithmic values of the cumulative *β_mlh_* and stepwise protonation constants *K_mlh_* of the disaccharide ligand **9** determined by potentiometric and spectrophotometric titrations. **M** designating the metal ion, **L**, the ligand, and **H**, the proton; *m*, *l*, *h* indicating the stoichiometry in the notation of the complex according to the notation: $M_{m}L_{l}H_{h}$. For the ligand alone, *m = 0*. Charges are omitted for clarity.

		log *β_mlh_*	
Equilibrium Reaction	mlh	Potentiometry	Spectrophotometry
${L+3H⇌LH}_{3}$	013	18.51	18.16
${L+2H⇌LH}_{2}$	012	16.04	16.01
L+H⇌LH	011	8.44	8.49
		**log *K_mlh_***	
**Equilibrium reaction**	**mlh**	**Potentiometry**	**Spectrophotometry**
${{LH}_{2}+H⇌LH}_{3}$	013	2.47	2.15
${LH+H⇌LH}_{2}$	012	7.60	7.52
L+H⇌LH	011	8.44	8.49

I = 0.1 M (NMe_4_Cl); T = 298 K; *pK_w_* = 13.78. Values of *K_LH_^h^* were calculated from Equation (6).

**Table 2:** UV–visible spectral data for the disaccharide ligand **9**.

		λ = 250 nm
Species	mlh	ε (mol^−1^ L cm^−1^)
L	010	333.6
LH	011	278.4
${LH}_{2}$	012	104.4
${LH}_{3}$	013	105.5

**Table 3:** Logarithmic values of the cumulative *β_mlh_* and Stepwise Stability Constants *K_mlh_* for the cobalt(II):**9** complex determined by potentiometric and spectrophotometric titrations. Charges are omitted for clarity.

		log *β_mlh_*			
**Equilibrium reaction**	**mlh**	**Potentiometry**	**Spectrophotometry**		
			**1:1**	**1.5:1**	**2:1**
${Co+L+2H⇌CoLH}_{2}$	112	19.17	20.08	19.08	19.09
Co+L+H⇌CoLH	111	16.53	16.90	16.29	16.15
Co+L⇌CoL	110	12.28	12.34	11.49	11.75
${Co+L⇌CoLH}_{-1}+H$	11−	4.48	4.60	4.50	4.46
${Co+L⇌CoLH}_{-2}+2H$	11−2	−6.18	−6.23	−5.99	−5.53
${2Co+L+2H⇌{Co}_{2}LH}_{2}$	212	22.23	22.00	22.08	21.89
$2Co+L+H⇌{Co}_{2}LH$	211	19.18	18.99	19.04	18.79
$2Co+L⇌{Co}_{2}L$	210	16.53	15.79	15.81	15.21
${2Co+L⇌{Co}_{2}LH}_{-1}+H$	21−1	12.28	12.44	11.78	11.52
${2Co+L⇌{Co}_{2}LH}_{-2}+2H$	21−2	4.43	4.19	2.03	3.07
${Co⇌CoH}_{-1}+H$	10−1	−8.43 ^†^			
${Co⇌CoH}_{-2}+2H$	10−2	−16.88 ^†^			
${Co⇌CoH}_{-3}+3H$	10−3	−21.9 ^†^			
		**log *K_mlh_***			
**Equilibrium reaction**	**mlh**	**Potentiometry**	**Spectrophotometry**		
			**1:1**	**1.5:1**	**2:1**
${CoLH+H⇌CoLH}_{2}$	112	2.64	3.18	2.79	2.94
CoL+H⇌CoLH	111	4.25	4.56	4.8	4.41
Co+L⇌CoL	110	12.28	12.34	11.49	11.75
${CoL⇌CoLH}_{-1}+H$	11−1	−7.8	−7.74	−6.99	−7.29
${{CoLH}_{-1}⇌CoLH}_{-2}+H$	11−2	−10.66	−10.83	−10.49	−9.99
${{Co}_{2}LH+H⇌{Co}_{2}LH}_{2}$	212	3.05	3.01	3.04	3.1
${Co}_{2}L+H⇌{Co}_{2}LH$	211	2.65	3.20	3.23	3.58
$2Co+L⇌{Co}_{2}L$	210	16.53	15.79	15.81	15.21
${Co}_{2}L{⇌{Co}_{2}LH}_{-1}+H$	21−1	−4.25	−3.35	−4.03	−3.69
${{{Co}_{2}LH}_{-1}⇌{Co}_{2}LH}_{-2}+H$	21−2	−7.85	−8.25	−9.75	−8.45

I = 0.1 N (NMe_4_Cl); T = 298 K; *pK_w_* = 13.78. Values in parenthesis are standard deviations in the last significant figures. Values of *K_mlh_* calculated from Equation (6). ^†^ This study to compare with references [26,32,33,34,35].

**Table 4:** UV–visible spectral data for the cobalt(II):**9** complex 1:1.

		λ = 250 nm
Species	mlh	ε (mol^−1^ L cm^−1^)
${CoLH}_{2}$	012	273.9
CoLH	011	1158.6
CoL	010	700.4
${CoLH}_{-1}$	01−1	662.6
${CoLH}_{-2}$	01−2	645.2
${{Co}_{2}LH}_{-1}$	21−1	309.7

**Table 5:** Logarithmic values of the cumulative *β_lh_* and stepwise protonation constants *K_lh_* of *Pipes* and *Mes* and determined values of the cumulative *β_mlh_* and stepwise stability constants *K_mlh_* for the cobalt(II)–buffer complexes by potentiometric titrations at 298 K, I = 0.1 N (NMe_4_Cl), *pK_w_* = 13.68. Charges are omitted for clarity. * Values of $\log\beta_{mlh}^{*}$ calculated from Equation (6).

log *K_mlh_*					
***Pipes***	**mlh**		***Mes***	**mlh**	
$PipesH+H⇌{PipesH}_{2}$	012	2.55			
Pipes+H⇌PipesH	011	6.62	Mes+H⇌MesH	011	5.98
**log *β_mlh_***					
Co+Pipes⇌CoPipes log *β_CoPipes_*	110	2.2	Co+Mes⇌CoMes log *β_CoMes_*	110	1.9
$Co+Pipes⇌CoPipesH_{-2}+2H$ log *β_CoPipesH-2_*	11-2 *	* −14.5	$Co+Mes⇌CoMesH_{-2}+2H$ log *β_CoMesH-2_*	11-2 *	* −15.2
$Co+Pipes+2OH⇌{CoPipes(OH)}_{2}$ log *_βCoPipes(OH)2_*	11-2	12.9	$Co+Mes+2OH⇌{CoMes(OH)}_{2}$ log *β_CoMes(OH)2_*	11-2	12.1
$CoPipes+2OH⇌{CoPipes(OH)}_{2}$ log *K_CoPipes(OH)2_*	11-2	10.7	$CoMes+2OH⇌{CoMes(OH)}_{2}$ log *K_CoMes(OH)2_*	11-2	10.2

**Table 6:** Experimental concentrations (mM) used in the direct (experiments **1** to **3**) and inverse titrations (experiment **4**).

*Pipes*	Experiment	1	2	3	4
**Syringe**	[cobalt(II)]	14	7	2	0
	[9]	0	0	0	1.05
**Cell**	[cobalt(II)]	0	0	0	0.15
	[9]	0.92	0.44	0.144	0
***Mes***					
**Syringe**	[cobalt(II)]	14	7	3.5	0
	[9]	0	0	0	0.78
**Cell**	[cobalt(II)]	0	0	0	0.15
	[9]	0.78	0.39	0.20	0

## Data Availability

Data are contained within this article and Supplementary Materials.

## Supplementary Materials

The following supporting information can be downloaded at: https://www.mdpi.com/article/10.3390/molecules30153263/s1. S1. NMR study. S2. Structure of usual ligands and log *K*. S3. ICP-AES study. S4. Analysis of complexes Co(II):**9**. S5. Desorption experiments.
