# Supplementary material for: A Comprehensive Study of the Cobalt(II) Chelation Mechanism by an Iminodiacetate-Decorated Disaccharide Ligand
Source: Molecules. 2025 Aug 4;30(15):3263. doi: 10.3390/molecules30153263 (PMC12348390; doi:10.3390/molecules30153263)
Supplement: Supplementary file 1 [file molecules-30-03263-s001.zip › molecules-3763125-supplementary.pdf]

SUPPORTING INFORMATION

A Comprehensive Study of Cobalt(II) Chelation Mechanism by an Iminodiacetate-decorated Disaccharide Ligand

Cécile Barbot[1], Laura Gouriou[1], Mélanie Mignot[1], Muriel Sebban[1], Ping Zhang[2], David Landy[3], Chang-Chun Ling[2], and Géraldine Gouhier*[1]

[1]*Normandie Université, COBRA UMR 6014, FR 3038, INSA Rouen, CNRS, IRCOF, France*
[2]*UCEIV, UR4492 Université du Littoral Côte d'Opale, Dunkerque, France*
[3]*Department of Chemistry, University of Calgary, Calgary Alberta T2N 1N4, Canada*
e-mail: Geraldine.gouhier@univ-rouen.fr

Table of Contents

The synthesis of Ligand **9** has been described in the publication: Champagne, P.-L.; Barbot, C.; Zhang, P.; Han, X.; Gaamoussi, I.; Hubert-Roux, M.; Bertolesi, G. E.; Gouhier, G.; Ling C.-C. Synthesis and Unprecedented Complexation Properties of β-Cyclodextrin-Based Ligand for Lanthanide Ions. *Inorg. Chem.* **2018,** *57,* 8964-8977.
https://doi.org/10.1021/acs.inorgchem.8b00937

S1. NMR study
S2. Structure of usual Ligands and logK
S3. ICP-AES Study
S4. Analysis of complexes Co(II):9
S5. Desorption experiments

S1. NMR Study

Acquisition parameters were first optimized using a reference sample (Co:Bipyridine complex) described in literature [M. Lehr *et al.*, Angew. Chem. Int. Ed., **2020**, *59*, 19344-19351]. ^{1}H and ^{13}C (without ^{1}H decoupling) NMR spectra were acquired with a large spectral width (SW): 150 ppm and 300 ppm for ^{1}H, and 300 and 1000 ppm for ^{13}C. Small relaxation delays (D1=1 ms for ^{1}H and D1=50 ms for ^{13}C) and a large number of transients (NS=1000 scans for ^{1}H and NS=10000 scans for ^{13}C) were used.
^{13}C spectra with ^{1}H decoupling NMR spectra were also recorded using D1=5 s, NS=20000 scans, SW=355 ppm centered on O1=100 ppm). Finally, ^{1}H spectrum with a presaturation of the residual HDO signal was acquired using SW = 300 ppm (centered on O1=4.70 ppm, water resonance), D1=1 s, and NS=1000 scans.

NMR data

Ligand 9

^{1}H NMR (400 MHz, D_2O, 298K): δ (ppm) 7.98 (s, 1H, H triazole), 7.94 (s, 1H, H triazole), 5.30 (d, *J* = 3.8 Hz, H1), 4.81 (dd, *J* = 14.9; 1.8 Hz, H6a), 4.59-4.53 (m, 2H H6b, H1'), 4.15-4.00 (m, 5H, H6a', H8, H8', H6b', H5), 3.86-3.64 (m, 5H, H5', H3', H8, H8', H3), 3.51 (dd, *J* = 10.0; 3.8 Hz, H2), 3.44 (dd, *J* = 9.9; 3.8 Hz, H2'), 3.30-3.14 (m, 10H, H4', H9, H9', H4), 2.89 (s, 3H, OMe).

^{13}C NMR (100 MHz, D_2O, 298K): δ (ppm) 127.55 (CH triazole), 126.96 (CH triazole), 100.20, 98,69 (C1, C1'), 79.62 (C4'), 73.23 (C3'), 72.44 (C3), 71.51 (C2), 71.44 (C5), 70.67 (C4, C2'), 68.11 (C5'), 57.20, 56.94 (C9, C9'), 54.60 (OMe), 51.11, 50.54 (C6, C6'), 47.98, 47.90 (C8, C8')

Co(II):9 1:1

^{1}H NMR (400 MHz, D_2O, 298K): δ (ppm) 125.52, 119.03, 89.35, 71.91, 54.42, 47.46, 33.25, 32.13, 18.32, 14.16, 12.87, 12.05, 10.85, 8.08, 7.50, 6.71, 6.05, 5.84, 5.42, 4.06, 3.49, 3.33, 2.69, 2.27, 2.17, 1.82, 1.70, 0.07, -3.38, -4.97, -6.38, -11.41, -17.17, -35.22, -42.42, -60.61, -85.10, -97.51.

^{13}C NMR (100 MHz, D_2O, 298K): δ (ppm) 198.36, 190.52, 106.51, 99.98, 98.78, 97.56, 92.08, 91.39, 78.79, 77.89, 76.18, 75.25, 74.32, 73.18, 72.02, 70.77, 70.06, 66.87, 64.38, 52.80.

Co(II):9 2:1

^{1}H NMR (400 MHz, D_2O, 298K): δ (ppm) 125.22, 124.72, 116.18, 88.24, 70.51, 57.96, 56.65, 53.31, 48.25, 46.67, 42.23, 39.89, 32.57, 10.87, 8.97, 8.03, 6.78, 4.08, 3.38, 3.25, 3.06, 2.69, 2.51, 2.25, 1.98, 1.80, 1.60, 1.38, 0.90, 0.68, 0.55, 0.06, -0.42, -1.12, -3.17, -3.34, -3.87, -4.91, -6.27, -11.29, -17.04, -34.91, -42.18, -61.36, -83.67, -95.37.

^{13}C NMR (100 MHz, D_2O, 298K): δ (ppm) 189.87, 181.40, 155.74, 121.28, 118.00, 100.81, 99.98, 91.96, 87.49, 77.96, 74.99, 71.73, 70.05, 68.12, 66,84, 60.13, 59.68.

S2. Structure of usual Ligands and logK

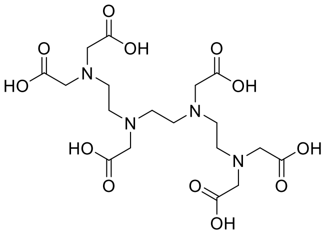

Triethylenetetramine-N,N,N',N'',N''',N'''-hexaaceti(
acid
TTHA

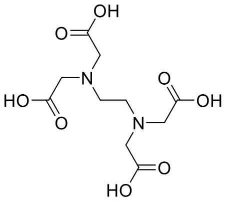

Ethylenediaminetetraacetic
acid
EDTA

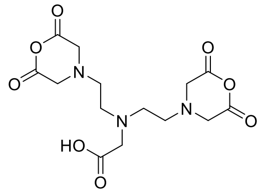

Diethylenetriaminepentaacetic dianhydride
DTPA

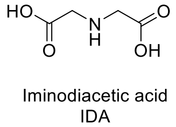

Iminodiacetic acid
IDA

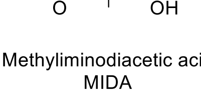

Methyliminodiacetic acid
MIDA

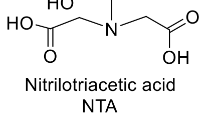

Nitrilotriacetic acid
NTA

Figure S1. Structures of ligands IDA, MIDA, DTPA, TTHA, EDTA, and NTA

IUPAC Names:

IDA: iminodiacetic acid

MIDA: *N*-methylimninodiacetic acid

DTPA: Diethylenetriamine-*N*,*N*,*N*′,*N*″,*N*″-pentaacetic dianhydride

TTHA: Triethylenetetramine-*N*,*N*,*N*′,*N*″,*N*‴,*N*‴-hexacetic acid

EDTA: Ethylenediamine-*N*,*N*,*N*′,*N*′-tetraacetic acid

NTA: 2,2',2''-Nitrilotriacetic acid
Charges are omitted for simplicity.

Table S1. Conditions reported and logK for IDA and MIDA Ligands[7a, unless other reference mentioned]

				log*K*[7a]			
Cation	Equilibrium reaction	I (mol/L)	t (°C)	IDA	I (mol/L)	t (°C)	MIDA
H	$L + H \rightleftharpoons LH$	0	25	9.79[7k]	0	25	10.01[7k]
		0.1; NMe_4Br	25	9.45 (0.01)			
		0.1; KNO_3	20	9.45 (0.02)	0.1; KCl	20	9.65 (0.07)
		0.1; KNO_3	25	9.47[7e]			
		0.1; KNO_3/Cl	25	9.32 (0.02)	0.1; KNO_3/Cl	25	9.59 (0.02)
		0.2; KCl	25	9.29 (0.03)			
		0.5; KNO_3	25	9.25 (0.06)	0.5; KNO_3	25	9.46 (0.03)
		1.0; KNO_3/Cl	25	9.27 (0.03)			
		0.1; $NaClO_4$	35	9.25 (0.06)			
		0.5; $NaClO_4$	25	9.22 (0.05)	0.5; $NaClO_4$	25	9.43 (0.03)
		1.0; $NaClO_4$	20	9.33 (0.08)	1.0; $NaClO_4$	25	9.48 (0.06)
		1.0; $NaClO_4$	25	9.29 (0.05)	2.0; KNO_3	35	9.50[7k]
	$LH + H \rightleftharpoons LH_2$	0	25	2.84[7k]	0	25	2.59[7k]
		0.1; KNO_3/Cl	25	2.60 (0.03)			
		0.1; KNO_3	25	2.68[7e]	0,1; KCl	20	2.12 (0.09)
		0.2; KCl	25	2.54 (0.04)			
		0.5; KNO_3	25	2.53 (0.06)	0.5; KNO_3	25	2.32 (0.03)
		1.0; KNO_3/Cl	25	2.60 (0.03)			
		0.1; $NaClO_4$	25	2.70 (0.07)			
		0.1; $NaClO_4$	35	2.66 (0.03)			
		0.5; $NaClO_4$	25	2.58 (0.02)	0.5; $NaClO_4$	25	2.28 (0.02)
		1.0; $NaClO_4$	20	2.64 (0.03)			
		1.0; $NaNO_3$	25	2.65 (0.04)			
		1.0; $NaClO_4$	25	2.58 (0.03)	1; $NaClO_4$	25	2.4 (0.1)
	$LH_2 + H \rightleftharpoons LH_3$	0	25	1.8[7k]	0	25	1.9[7k]
		1.0; KNO_3/Cl	25	1.82 (0.06)	0.5; KNO_3	25	1.4 (0.1)
		0.5; $NaClO_4$	20	1.8 (0.1)			

		0.5; $NaClO_4$	25	1.79 (0.03)			
		1.0; $NaClO_4$	25	1.87 (0.02)	1.0; $NaClO_4$	25	1.6 (0.1)
$Co(II)$	$Co + L \rightleftharpoons CoL$	0	25	7.82[7k]	0	25	8.5[7k]
		0.1; KNO_3	25	6.97 (0.05), 7[7b]			
		1.0, $NaClO_4$	25	6.54			
		0.1; KCl	30	6.95 (0.02)	0.1; KCl	20	7.6 (0.1)
	$Co + 2L \rightleftharpoons CoL_2$	0	25	13.2[7k]	0	25	14.70[7k]
		0.1; KNO_3	20	12.3 (0.2)			
		0.1; KCl	30	12.3 (0.1), 12;3[7b]	0.1; KCl	20	13.9 (0.1), 13.8[7k]
		1.0	25	11.8[7k]			

Table S2. Conditions tested and logK for DTPA and TTHA[7a, unless other reference mentioned]

				log*K*[7a]				
Cation	Equilibrium reaction	I (mol/L)	t (°C)	DTPA	Equilibrium reaction	I (mol/L)	t (°C)	TTHA
H	$L + H \rightleftharpoons LH$	0.1; KNO_3	20	10.58 (0.03)		0.1; KNO_3	20	10.65 (0.03)
		0.1; KNO_3	25	10.54 (0.03)		0.1; KNO_3	25	10.62 (0.02)
		0.15; $NaClO_4$	25	9.76 (0.02)		0.5; $NaClO_4$	25	9.73 (0.05)
		0.15; NaCl	37	9.67 (0.02)		1.0; $NaClO_4$	25	9.43 (0.03)
		1.0; NMe_4Cl	20	10.46 (0.03)		0.1; NMe_4NO_3	25	10.63 (0.05)
		1.0; KCl	25	10.06 (0.03)		1.0; $NaNO_3$	25	9.39 (0.02)
		1.0; NaCl	20	9.48 (0.03)				
		0	25	11.52[7c], 11.55[7k]		0	25	11.71[7c]
	$LH + H \rightleftharpoons LH_2$	0.1; KNO_3	20	8.60 (0.05)		0.1; KNO_3	20	9.54 (0.03)
		0.1; KNO_3	25	8.56 (0.01)		0.1; KNO_3	25	9.54 (0.03)
		0.15; $NaClO_4$	25	8.33 (0.03)		0.1; NMe_4NO_3	25	9.46 (0.02)
		0.15; NaCl	37	8.27 (0.03)		0.5; $NaClO_4$	25	8.76 (0.05)
		1.0; NMe_4Cl	20	8.41 (0.03)		1.0; $NaClO_4$	25	8.69 (0.03)
		1.0; KCl	25	8.32 (0.03)		1.0; $NaNO_3$	25	8.74 (0.03)
		1.0; NaCl	20	8.26 (0.03)				
		0	25	9.36[7c], 9.5[7k]		0	25	10.42[7c]
	$LH_2 + H \rightleftharpoons LH_3$	0.1; KNO_3	20	4.30 (0.03)		0.1; KNO_3	20	6.10 (0.02)

		0.1; KNO_3	25	4.30 (0.03)		0.1; KNO_3	25	6.15 (0.03)
		0.15; $NaClO_4$	25	4.18 (0.03)		0.1; NMe_4NO_3	25	6.11 (0.03)
		0.15; $NaClO_4$	37	4.15 (0.03)		0.5; $NaClO_4$	25	5.92 (0.05)
		1.0; NMe_4Cl	20	4.14 (0.03)		1.0; $NaClO_4$	25	6.00 (0.03)
		1.0; KCl	25	4.13 (0.03)		1.0; $NaNO_3$	25	5.88 (0.03)
		1.0; NaCl	20	4.19 (0.03)				
		0	25	4.83[7c], 4.92[7k]		0	25	6.83[7c]
	$LH_3 + H \rightleftharpoons LH_4$	0.1; KNO_3	20	2.58 (0.03)		0.1; KNO_3	20	4.03 (0.04)
		0.1; KNO_3	25	2.77 (0.05)		0.1; KNO_3	25	4.07 (0.03)
		0.15; $NaClO_4$	25	2.68 (0.03)		0.1; NMe_4NO_3	25	4.04 (0.04)
		0.15; $NaClO_4$	37	2.68 (0.03)		0.5; $NaClO_4$	25	3.94 (0.05)
		1.0; NMe_4Cl	20	2.7 (0.1))		1.0; $NaClO_4$	25	3.99 (0.05)
		1.0 KCl	25	2.5 (0.1)		1.0; $NaNO_3$	25	3.97 (0.03)
		1.0 NaCl	20	2.5 (0.1)				
		0	25	3.13[7c]		0	25	4.61[7c]
				log*K*[7a]				
**Cation**	**Equilibrium reaction**	**I (mol/L)**	**t (°C)**	**DTPA**	**Equilibrium reaction**	**I (mol/L)**	**t (°C)**	**TTHA**
	$LH_4 + H \rightleftharpoons LH_5$	0.1; KNO_3	20	1.8 (0.1)		0.1; KNO_3	20	2.7 (0.1)
		0.1; KNO_3	25	2.0 (0.1)		0.1; KNO_3	25	2.79 (0.07)
		0.15; $NaClO_4$	25	2.0 (0.1)		0.1; NMe_4NO_3	25	2.75 (0.07)
		0.15; NaCl	37	2.1 (0.1)		0.5; $NaClO_4$	25	2.8 (0.1)
		1.0; NMe_4Cl	20	2.2 (0.1)		1.0; $NaClO_4$	25	2.7 (0.1)
		1.0 KCl	25	2.3 (0.1)		1.0; $NaNO_3$	25	2.6 (0.1)
		1.0 NaCl	20	1.9 (0.1)				
		0	25	2.35[7c]		0		3.20[7c]
	$LH_5 + H \rightleftharpoons LH_6$	1.0; Na/$HClO_4$	20	1.2 (0.2)		0.1; KNO_3	20	2.3 (0.1)
		1.0; K/HCl	25	1.7 (0.2)		0.1; KNO_3	25	2.2 (0.1)
						0.1; NMe_4NO_3	25	2.34 (0.07)
						0.5; $NaClO_4$	25	2.3 (0.1)
						1.0; $NaClO_4$	25	2.3 (0.1)
						1.0; $NaNO_3$	25	2.2 (0.1)

						0	25	2.42[6c]
	$LH_6 + H \rightleftharpoons LH_7$					0.1; KNO_3	25	1.8 (0.1)
	$LH_7 + H \rightleftharpoons LH_8$					0.1; KNO_3	25	1.5 (0.1)
$Co(II)$	$Co + L \rightleftharpoons CoL$	0	25	21.29[7k]	$Co + L \rightleftharpoons CoL$	0.1	15	18.4[7b], 20.4[7f]
		0.1; $NaNO_3$	20	19.3 (0.1), 19.1[7b]				
	$CoL + H \rightleftharpoons CoLH$	0.1; $NaNO_3$	20	4.72 (0.05)	$CoL + H \rightleftharpoons CoLH$	0.1; KNO_3	25	8.2 (0.2)
	$CoL + Co \rightleftharpoons Co_2L$	0.1; $NaNO_3$	20	3.5 (0.1)	$Co_2L + H \rightleftharpoons Co_2LH$	0.1; KNO_3	25	3.0 (0.2)
					$Co_2LH + H \rightleftharpoons Co_2LH_2$	0.1; KNO_3	25	2.6 (0.2)

Table S3. Conditions tested and logK for EDTA and NTA[7a, unless other reference mentioned]

					log7[6k]			
Cation		Equilibrium reaction	I (mol/L)	t (°C)	EDTA	I (mol/L)	t (°C)	NTA
H		$L + H \rightleftharpoons LH$	0	25	10.39[7d]	0	25	11.09[7d]
			0	25	10.948	0	20	10.334
			0	25	9.65[7g] (0.01)	0	25	11.45[7g] (0.02)
			0.1; NaCl	25	10.45[7g] (0.01)	0.1; NaCl	25	9.58[7g] (0.02)
			0.1; NaCl	25	9.52	0.1; NaCl	25	9.46
			0.15; NaCl	37	9.38	0.15; NaCl	37	9.25
			0.5; NaCl	25	8.86	0.5; NaCl	25	8.94
			1.0; NaCl	25	8.73	1.0; NaCl	25	8.95
			0.1; KCl	25	10.26[7j]	0.1; KCl	25	9.59[6i]
			0.1; KCl	25	10.19	0.1; KCl	25	9.66
			0.15; KCl	37	10.02	0.15; KCl	37	9.55
			0.5; KCl	25	9.62	0.5; KCl	25	9.31
			0.5; KNO_3	25	10.50[7i](0.02)	0.5; KNO_3	25	9.57[7i] (0.06)
						2.0; KNO_3	25	9.39
			1.0; KCl	25	9.86	1.0; KCl	25	9.34
			0.1; NMe_4Cl	25	10.37	0.1; NMe_4Cl	25	9.84
			0.5; NMe_4Cl	25	10.23			
			0.15; NMe_4Cl	37	10.21	0.15; NMe_4Cl	37	9.72
			1.0; NMe_4Cl	25	10.2			
						2.0; $NaClO_4$	25	9.08
			3.0; $NaClO_4$	25	9.05	3.0; $NaClO_4$	25	9.35
		$LH + H \rightleftharpoons LH_2$	0	25	6.72[7d]	0	20	2.94[7d]
			0	25	6,273			
			0	25	6.74[7g] (0.01)	0	25	3.09[7g] (0.22)
			0.1; NaCl	25	6.10[7g] (0.01)	0	25	2.67[7g] (0.22)
			0.1; KCl	25	6.16, 6.13	0.1; KCl	25	2.52[7h]
			0.15	37	6.03[7l]	0.15	37	2.50
			0.5; KNO_3	25	6.26[7i] (0.06)	0.5; KNO_3	25	2.64[7i] (0.04)

			0.5	25	6.10	0.5	25	2.31[7l]
			1.0	25	6.19	1.0	25	2.28
						2.0; $NaClO_4$	25	2.46
						2.0; KNO_3	25	2.45
			3.0	25	7.02[7l]	3.0; $NaClO_4$	25	2.64
					log[7k]			
**Cation**		**Equilibrium reaction**	**I (mol/L)**	**t (°C)**	**EDTA**	**I (mol/L)**	**t (°C)**	**NTA**
		$LH_2 + H \rightleftharpoons LH_3$	0	25	3.02[7d]	0	25	1.97[7d]
			0	25	2.96[7g] (0.02)	0	25	1.71[7g] (0.18)
			0.1; NaCl	25	2.50[7g] (0.02)	0.1; NaCl	25	1.50[7g] (0.18)
			0.1; KCl	25	2.67[7j]	0.1; KCl	25	1.50[7h]
			0.1	25	2.69			
			0.5; KNO_3	25	2.68[7i] (0.04)	0.5; KNO_3	25	1.57[7i] (0.06)
		$LH_3 + H \rightleftharpoons LH_4$	0	25	2.12[7d]			
			0	25	1.36[7g] (0.06)			
			0.1; NaCl	25	1.12[7g] (0.06)			
			0.1; KCl	25	2.0[7j]			
			0.15	37	2.79			
			0.5; KNO_3	25	1.89[7h] (0.06)			
			1.0	25	2.52			
			3.0	25	2.54			
		$LH_4 + H \rightleftharpoons LH_5$	0.1	25	2.00			
			0.15	37	2.05			
			1.0	25	2.02			
			3	25	2.20			
$Co(II)$		$Co + L \rightleftharpoons CoL$	0.1; KCl	25	16.4[7b], 16.45	0.1; KCl	25	10.3[7b], 10.38
		$CoL + H \rightleftharpoons CoLH$	0.1, 1.0	25	3.0			
		$Co(OH)L + H \rightleftharpoons CoL$				0.1; KCl	25	10.8
		$Co + 2L \rightleftharpoons CoL_2$				0.1; KCl	25	14.3[7b], 14.33

S3. ICP-AES Study

Preparation of standards

A stock solution of 1 g/L of Co(II) in 2% HNO_3 was prepared using Cobalt(II) chloride hexahydrate salt (Sigma Aldrich). Standard solutions were prepared by dilution, while maintaining a 2% (v/v) nitric acid concentration. For the calibration, this solution was further diluted to 10- and 100-fold with 2% HNO_3.

Table S4. Experimental conditions

iCAP 6000 ICP-AES			
**Radio frequency power (W)**	1150	**Sample uptake delay (s)**	30
**Plasma gas flow rate (L/min)**	14	**Replicates**	3
**Auxiliary argon flow rate (L/min)**	0.5	**Rinse time (s)**	30
**Nebulizer gas flow rate (L/min)**	0.5	**Sampler**	Manual
**Purge gas (nitrogen) flow rate (L/min)**	12	**Chamber**	Cyclonic
**Peristatic pump rate (rpm)**	50	**Nebulizer type**	Concentric

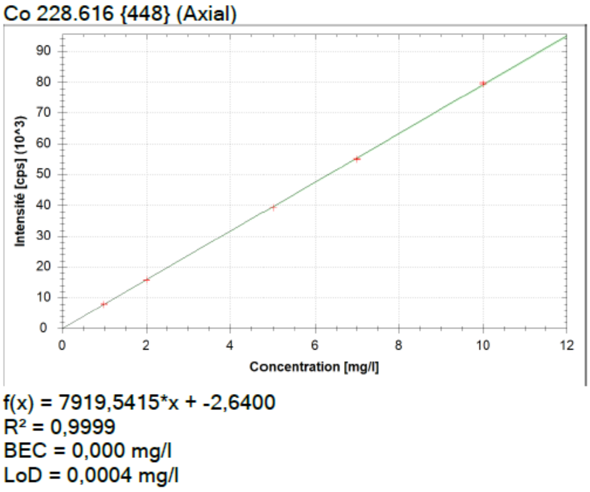

Figure S2. Calibration curve for ICP-MS experiment

S4. Analysis of Complexes Co(II):9

HRMS experiments of the complexes were performed using a quadrupole time-of-flight (Q-TOF) mass spectrometer (Synapt G2 HDMS, Waters Corp., Manchester, UK) fitted with a lockspray ESI source. The protonated molecule of leucine enkephalin (*m/z* 554.2615) was used as an internal standard for the accurate mass measurements. Experiments were achieved in positive or negative ion modes.

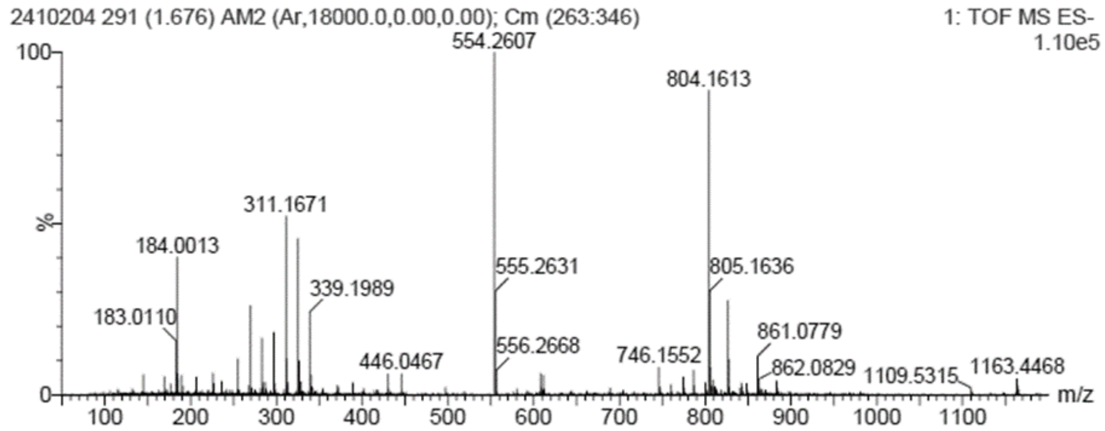

Figure S3. ESI HRMS spectrum in negative ion mode of compound Co(II):**9** 1:1.

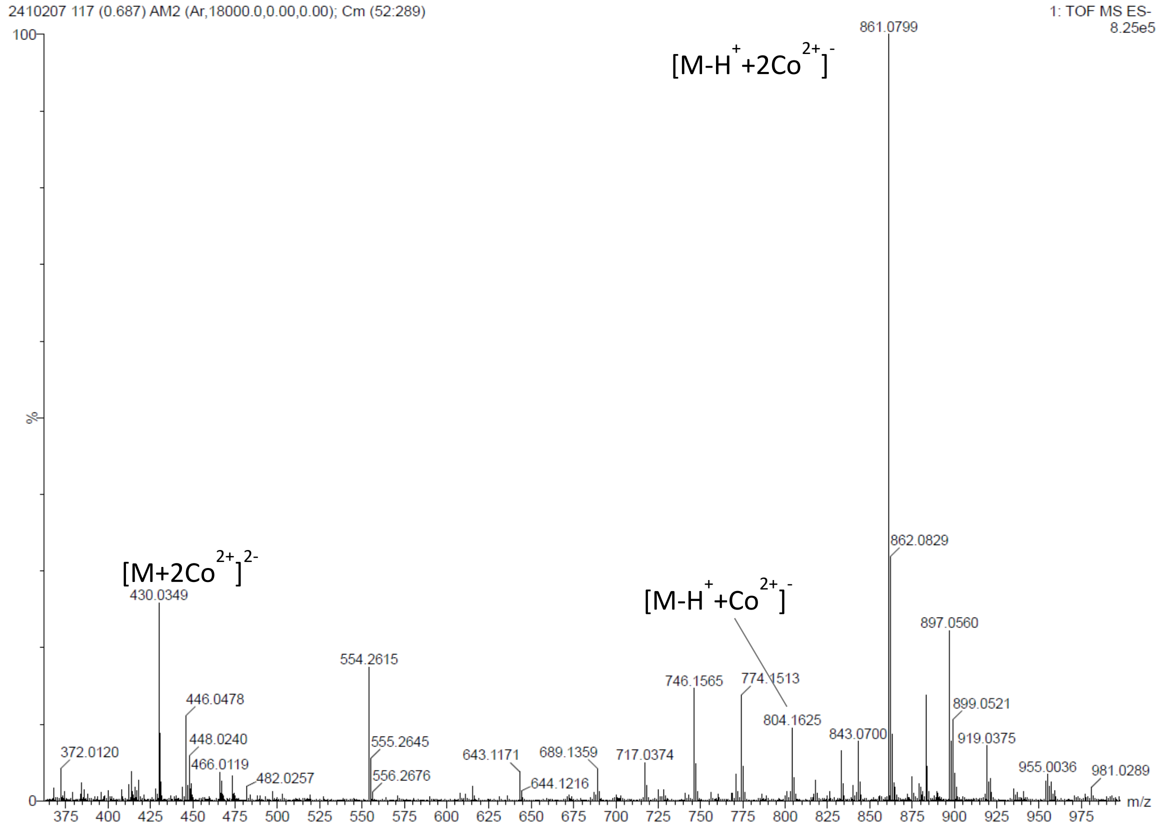

Figure S4. ESI HRMS spectrum in negative ion mode of compound Co(II):**9** 2:1.

S5. Desorption experiments

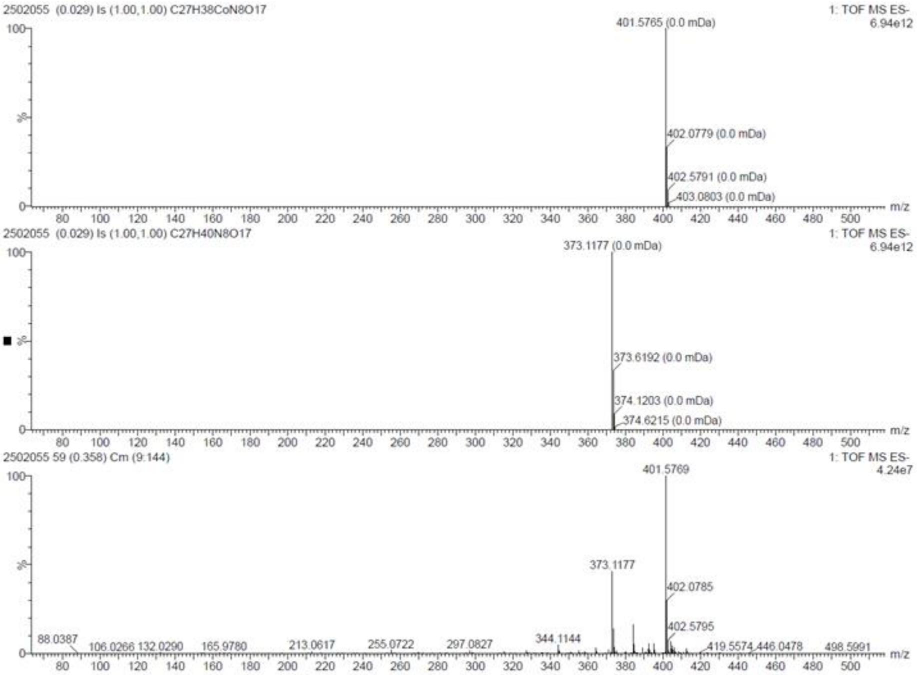

Figure S5. ESI-MS spectra of Co(II):**9** 2:1 complex after desorption on Chelex®

Table S5. HRMS attributions

Complexes	Ion ESI-	Calculated mass	Found mass	[M+X] species	Species ratio	Ratio ICP-AES
Co:**9** 1:1	$C_{27}H_{37}N_8O_{17}Co^-$	804.1609	804.1622	[ligand **9**-H+Co]$^-$	1:1	1:0.82±0.02
Co:**9** 1:2	$C_{27}H_{36}CoN_8O_{17}^{2-}$	401.5770	401.5765	[ligand **9**-4H+Co]$^{2-}$	1:1	1:1.6±0.05
	$C_{27}H_{34}Co_2N_8O_{17}^{2-}$	430.0358	430.0353	[ligand **9**-6H+2Co]$^{2-}$	1:2	
	$C_{27}H_{37}CoN_8O_{17}^-$	804.1614	804.1624	[ligand **9**+Co]	1:1	
	$C_{54}H_{72}Co_3N_{16}O_{34}^{2-}$	832.6202	832.6220	[2 ligand **9**+3Co]$^{2-}$	1:1.5	
	$C_{27}H_{35}Co_2N_8O_{17}^-$	861.0789	861.0815	[ligand **9**+2Co]$^-$	1:2	
Co:**9** 1:2 decomplexed	$C_{27}H_{38}N_8O_{17}^{2-}$	373.1183	373.1177	[ligand **9**-2H]$^{2-}$	1:0	1:0.3±<0.001
	$C_{27}H_{36}CoN_8O_{17}^{2-}$	401.5770	401.5765	[ligand **9**-4H+Co]$^{2-}$	1:1	
